# Chemical Characterization and Bio-Screening of Neuroprotective Potential of Brazilian Brown Seaweed *Canistrocarpus cervicornis* in 6-OHDA-Induced Neurotoxicity Model

**DOI:** 10.3390/antiox14121403

**Published:** 2025-11-25

**Authors:** Thalisia Cunha dos Santos, Johana Marcela Concha Obando, Joana Silva, Ana Luíza Vidal Pimentel Santos, Roberto Carlos Campos Martins, Diana Negrão Cavalcanti, Rui Pedrosa, Celso Alves

**Affiliations:** 1 Postgraduate Program in Chemistry of Natural Products, Instituto de Pesquisa em Produtos Naturais Walter Mors, Universidade Federal do Rio de Janeiro, Rio de Janeiro 21941-599, Brazil; roberto@ippn.ufrj.br; 2 Seallg—Solutions, Innovation, and Products, Startup, Bioeconomy Living Lab and Science and Technology-Based, Startup Incubators in the Ribeira Valley (Aquário de Ideias), Registro 11900-000, Brazil; johanamarcela@seallg.com; 3 National Institute of Science and Technology (INCT) in Nanotechnology for Sustainable Agriculture (INCTNanoAgro), Coordenação de Aperfeiçoamento de Pessoal de Nível Superior, Sorocaba 18087-180, Brazil; 4 MARE-Marine and Environmental Sciences Centre & ARNET, ESTM, Polytechnic University of Leiria, 2520-630 Peniche, Portugal; joana.m.silva@ipleiria.pt (J.S.); rui.pedrosa@ipleiria.pt (R.P.); 5 Faculty of Pharmacy, Universidade Federal do Rio de Janeiro, Rio de Janeiro 21941-853, Brazil; alvpsantos@ufrj.br; 6 Postgraduate Program in Marine Biology and Coastal Ecosystems, Instituto de Biologia, Universidade Federal Fluminense, Niterói 24210-201, Brazil; dncavalcanti@id.uff.br

**Keywords:** bioproducts, Dictyotaceae, neuroprotection, SH-SY5Y, neurological diseases, marine natural products, oxidative stress, mitochondrial disfunction, Apoptosis

## Abstract

Brazilian native seaweed *Canistrocarpus cervicornis* (Ochrophyta, Dictyotaceae) is recognized for its chemodiversity, particularly cyclic diterpenes and polysaccharides, yet its relevance to neurological disorders remains unexplored. This study evaluated the neuroprotective potential of a hydroethanolic extract (ECCH), its polar fraction (CCFPol), a dichloromethane extract (ECCD), and eight derived fractions (CCF1–2, CCF3, CCF4, CCF5–6, CCF7, CCF8–10, and CCF11–15). Cytotoxicity was evaluated in SH-SY5Y neuroblastoma cells, and neuroprotection was examined against 6-OHDA–induced toxicity. The mitochondrial membrane potential, ROS and H_2_O_2_ generation assays were conducted to explore the mechanisms underlying the observed effects. Among the key findings, the CCF3 fraction exhibited a high content (75.04%) of dolastane-type diterpenoids. Both CCFPol (100 µg/mL) and CCF3 (1 µg/mL) increased cell viability to 68.43 ± 4.60% and 60.61 ± 0.80%, respectively, compared with 6-OHDA–treated cells (50.70 ± 2.71%). Additionally, CCF3 and CCFPol reduced H_2_O_2_ levels (200.0 ± 18.19% and 195.5 ± 16.13%, respectively, vs. 6-OHDA-treated cells: 302.2 ± 17.07%) and lowered intracellular ROS (122.6 ± 22.7% and 129.6 ± 19.4%, respectively, vs. 6-OHDA-treated cells: 153.0 ± 32.7%). This is the first study to demonstrate the neuroprotective potential of the *C. cervicornis* in a 6-OHDA-induced neurotoxicity cellular model, contributing to the understanding of marine bioactive resources and their relevance for neurological research. Additional studies aimed at isolating the active constituents and clarifying their mechanisms of action will further strengthen and expand the biological relevance of this specie as source of neuroprotective agents.

## 1. Introduction

Neurological disorders, including Alzheimer’s disease (AD), Parkinson’s disease (PD), autism spectrum disorders (ASD), multiple sclerosis and others, represent significant global health challenges, impacting over three billion individuals worldwide and placing a burden on healthcare systems [1,2,3]. The clinical manifestations associated with these conditions are intricate and are not fully understood; however, previous studies suggest the involvement of neuroinflammation in the pathogenesis of these disorders, culminating in a distinctive array of symptoms (cognitive, sensory, and/or motor) primarily reflecting the main areas affected in each condition [4,5]. Previous reports have shown that toxic agents that cause oxidative stress may be involved in the pathophysiology of neurological diseases, especially PD, AD and ASD, due to the body’s ability to deal with excessive reactive oxygen species (ROS), which may vary between individuals due to genetic and age differences [5,6,7]. The parallels between the etiologies of these disorders suggest that biological models in which aspects of these diseases are present can be useful to search and develop new treatments involving the administration of neuroprotective agents [8]. The complexity of these conditions, coupled with the few effective treatment options available and targeted therapies, limited understanding of pathological mechanisms, and absence of specific biomarkers, underscores the need for research focused on developing neuroprotective agents capable of addressing various stages and severities [9,10,11].

The oceans are the largest habitat of living organisms in the world, sheltering an immense diversity of species and, therefore, constituting an interesting resource for obtaining bioactive compounds [12]. Among the vast marine biodiversity, macroalgae are considered exceptional sources of structurally diverse bioproducts with multiple biotechnological applications, and it is pertinent to encourage the development of studies on their potential in the treatment of neurological disorders [12,13,14].

The SH-SY5Y neuroblastoma cell line is one of the most widely used cell model to study neurological disorders and neurodevelopment due to low cost, ease of culture (feasibility), reproducibility and available literature [15,16,17,18,19]. Studies described the use of SH-SY5Y cells to evaluate the neuroprotective effect of natural products from seaweed [20,21,22,23]. The compound dieckol extracted from the brown seaweed *Ecklonia cava* exhibited potent antioxidant activity by preventing α-synuclein aggregation in the SH-SY5Y cell line [24]. The organic extracts from the brown seaweeds *Fucus serratus* Linnaeus and *Saccharina latissima* attenuate glutamate-induced toxicity in neuronal cells [25]. Fucoidan, a sulfated polysaccharide, suppresses the mitochondrial dysfunction of SH-SY5Y [26]. Additionally, fucoidans from *Sargassum hemiphyllum* reversed the 6-hydroxydopamine (6-OHDA)-induced neurotoxicity on SH-SY5Y cells. It is important to highlight that 6-OHDA is an oxidative metabolite of dopamine [22]. This neurotoxin can undergo extracellular auto-oxidation or intracellular enzymatic oxidation, yielding ROS and quinolinic products, and it is widely used in PD studies [27,28]. Extracts from *Saccorhiza polyschides*, *Sargassum muticum* and *Codium tomentosum* induced a remarkable increase in SH-SY5Y cells viability blunting the neurotoxicity mediated by 6-OHDA, suggesting their potential to be tested in neuroprotective therapeutic approaches [23].

Brown algae of the Dictyotaceae family (Phaeophyceae) hold a chemical arsenal that includes the production of cyclic diterpenes, sterols, fatty acids, and polysaccharides with significant biological activities, such as antibacterial, antiviral, antifungal, anti-inflammatory, among others [29,30,31,32,33]. Specifically, about the Dictyotaceae family, some studies reported the neuroprotective and anti-neuroinflammatory effects of extracts and diterpenes from the genus *Dictyota*. Xenicanes from *D. coriacea* demonstrated anti-neuroinflammatory effects on the oxygen-glucose deprivation and reperfusion (OGD/R)-induced SH-SY5Y cells and in vivo assay [34]. Fractions from *D. dichotoma* and *D. fasciola* exhibited acetylcholinesterase inhibitory activity [33]. Although diterpenoid compounds have been the primary focus of research in this family, polar fractions enriched in polysaccharide-type molecules warrant increased attention. Their hydrophilic character, high degree of sulfation or branching, and favorable water-solubility make them especially suited for neuroprotective investigations [8,14].

The species *Canistrocarpus cervicornis* (Dictyotaceae) is a producer of diterpenes with dolastane and secodolastane skeletons [35,36,37,38], which have demonstrated biological activities, such as antivirals acting against Human Immunodeficiency Virus Type 1 (HIV-1), Herpes Simplex Virus Type 1 (HSV-1), Zika Virus (ZIKV), and Chikungunya Virus (CHIKV) [39,40,41]; anticoagulant and antiplatelet [42]; and antiparasitic against the parasite *Leishmania amazonensis* in the promastigote, axenic amastigote and intracellular amastigote forms [43]. However, its neuroprotective potential remains unknown, offering an opportunity for the discovery of new bioactive compounds with potential for pharmacological applications in neurological disorders. Recognizing the potential of seaweeds from the Dictyotaceae family, this study presents the chemical characterization of extracts and fractions of *C. cervicornis* collected from the northern coast of Rio de Janeiro, Brazil (Arraial do Cabo). Specifically, it is reported the chemical characterization of its hydroethanolic and dichloromethane extracts, as well as eight enriched fractions, aiming to evaluate their neuroprotective potential. The study further investigates the mechanisms of action involved in protecting SH-SY5Y neuronal cells against 6-OHDA, with a focus on exploring its potential applications in neurological disorders.

## 2. Materials and Methods

### 2.1. Reagents

HPLC grade solvents dichloromethane (DCM), acetone, ethyl acetate (AcOEt), *n*-hexane, ethanol (EtOH) from Tedia (Fairfield, OH, USA) were used for extraction, sample preparation, chromatography methods and GC-MS. Fetal bovine serum (FBS), Dulbecco’s Modified Eagle’s Medium (DMEM), and antibiotic–antimycotic (pen/strep/fungiezone) solution were purchased from Biowest (Nuaillé, France). All remaining chemicals and reagents were acquired from Sigma-Aldrich (Carlsbad, CA, USA).

### 2.2. Sample Collection

**Figure 1:**
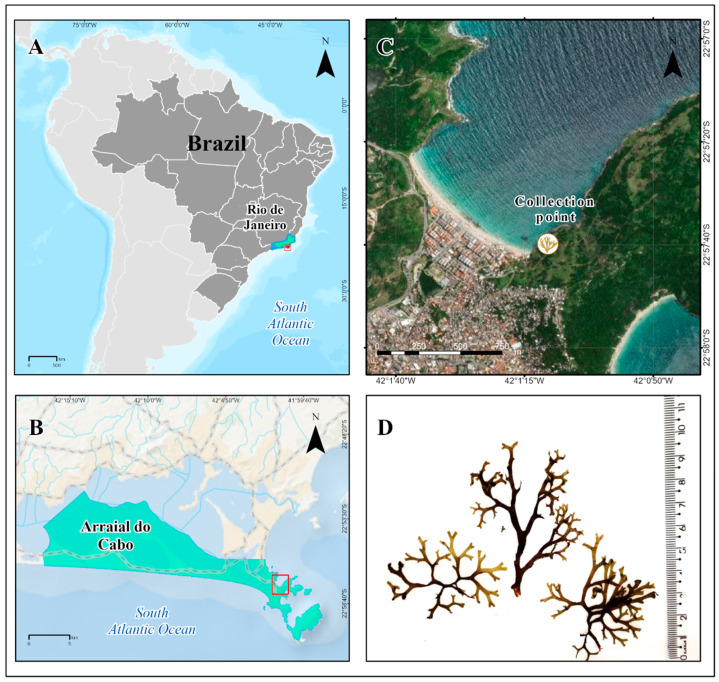
*Canistrocarpus cervicornis* collection site. (**A**) Map of Brazil highlighting the state of Rio de Janeiro. (**B**) Map showing the coastline of Arraial do Cabo (RJ, Brazil). (**C**) Spatial view of the collection site at Prainha Beach. (**D**) Representative photograph of *Canistrocarpus cervicornis*. Maps were generated by ArcGIS Pro (v. 3.2).

The brown seaweed *Canistrocarpus cervicornis* (Ochrophyta, Dictyotaceae) was collected in November 2023 at Prainha Beach in Arraial do Cabo (22°57′39.84″ S, 42°1′12.60″ W; Rio de Janeiro State, Brazil) (Figure 1) from shallow waters at a depth of ranging from 2.0 to 5.0 m through snorkeling. Access to the collected biomass is associated with the project registered in SISGEN under the code A133C0C. The visible epiphytic organisms were removed on site, and the collected algal biomass was kept in a cooler and transported to the Universidade Federal Fluminense for processing. Posteriorly, the seaweed was thoroughly washed with seawater and distilled water to remove epiphytes, sand, and debris. Then, the biomass was air-dried (around 28 to 30 °C, for 7 days) until all water had evaporated. In a mechanical grinder, the dried seaweed sample was ground into powder, and was kept in the freezer until extraction. The biomass was identified and deposited (Ref. HUNI6770) in the Herbário Prof. Jorge Pedro Pereira Carauta at Universidade Federal do Estado do Rio de Janeiro.

### 2.3. Seaweed Extraction and Fractionation

To explore the chemical diversity of *C. cervicornis* and evaluate compounds of distinct polarities, two extracts were prepared using solvents of different nature. The dichloromethane extract (ECCD) was designed to recover lipophilic constituents, while a hydroethanolic extract (ECCH) was obtained to concentrate more polar metabolites. The combination of both extraction approaches enables a broad assessment of the metabolomic profile of the species and supports the identification of bioactive fractions. Comprehensive methodological details regarding the extraction and fractionation steps are provided in the following paragraphs of this section.

The seaweed *C. cervicornis* (62.9 g) was subjected to exhaustive extraction with dichloromethane (1000 mL, 100%) for four weeks, with weekly renewal of the solvent, at room temperature and under light-protected conditions, resulting in the organic extract (ECCD). The ECCD (200 mg) was fractionated by column chromatography (25 cm length and 2 cm diameter) using silica gel (230–400 mesh; Sigma-Aldrich^®^) as the stationary phase, at a proportion of 1:30 (extract:silica, *w*/*w*). The extract was applied to the column in liquid form dissolved in the mobile phase used for elution. An isocratic elution was performed with a HEX:AcOEt (8:2, *v*/*v*) solvent system, yielding a total of 15 fractions. To enhance reproducibility and ensure consistent fractionation profiles, the column chromatography process was standardized and repeated 12 times under identical conditions, using the same initial extract in each run. Fractions were screened by thin layer chromatography (TLC) in TLC-plates of silica gel F254 (particle size of 10–12 µm, on aluminum support, Merck^®^, Darmstadt, Germany). The TLC plates were visualized by UV-irradiation at wavelengths of 254 and 356 nm. Additionally, the TLC plates were sprayed with acidified ceric sulfate solution (0.2%, *w*/*v*) (Sigma-Aldrich^®^) and subsequent heating step at 100 °C on a plate on an IKA^®^ C-MAG HS (Staufen, Germany) heating mantle. Fractions with the same retention factor (Rf) were pooled for further analysis by Nuclear Magnetic Resonance (NMR).

**Figure 2:**
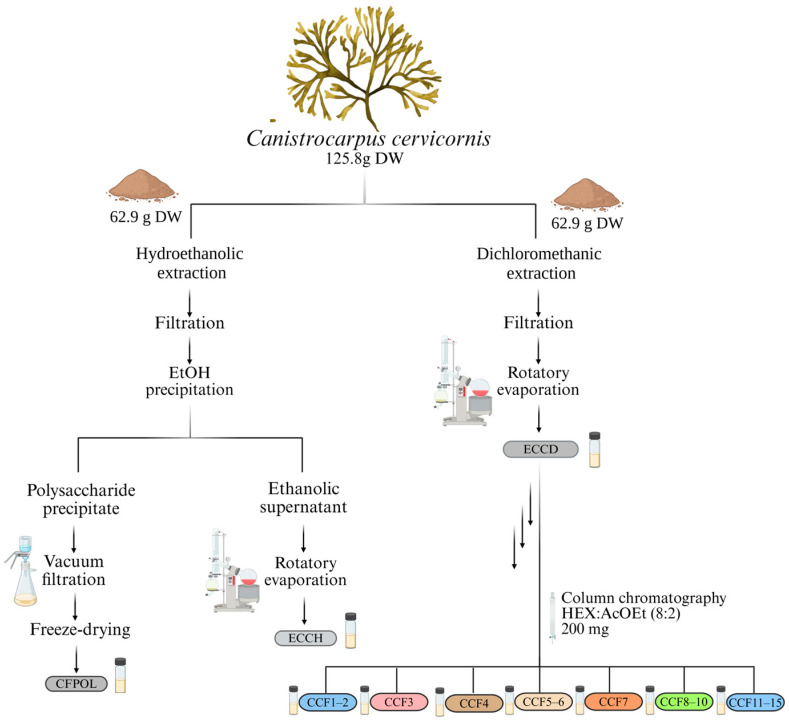
Extractions and fractionation workflow of *Canistrocarpus cervicornis*. Dried algal material was partitioned equally for solvent extraction under distinct polarity regimes. The hydroethanolic extract (ECCH) was obtained by maceration and vacuum filtration, followed by ethanol-induced precipitation to afford two fractions: a polysaccharide-rich precipitate (CCFPol). The dichloromethane extract (ECCD) was concentrated under reduced pressure and subjected to open-column chromatography on silica gel, eluted with a n-hexane/ethyl acetate (8:2, *v*/*v*) solvent system. This procedure generated eight fractions (CCF1–2, CCF3, CCF4, CCF5–6, CCF7, CCF8–10, CCF11–15). The three arrows, located on the right side of the scheme depict the sequential chromatographic steps used to obtain the fractions.

A hydroethanolic extract of *C. cervicornis* (62.9 g) was prepared using an EtOH:H_2_O solution (1000 mL, 70%, *v*/*v*) in the same period to obtain ECCH. The solvents were evaporated under reduced pressure using a rotary evaporator maintained at 40 °C (Büchi^®^ R-114, Flawil, Switzerland). Additionally, 100 mL of absolute EtOH were added to the hydroethanolic extract residue to induce polysaccharide precipitation. The EtOH-soluble supernatant was collected affording the ECCH crude extract and the resulting precipitate was abundantly washed with EtOH and acetone, vacuum filtered and subjected to lyophilization to ensure complete drying, obtaining a fraction rich in polysaccharides. The diagram of the process for obtaining extracts and fractions is shown in Figure 2.

### 2.4. Chemical Characterization

Gas Chromatography–Mass Spectrometry (GC–MS) analysis was performed using an Agilent 7890A/5973C system equipped with an HP5-MS column (30 m × 0.25 mm × 0.25 μm). For sample preparation, stock solutions were first prepared at 1000 µg/mL in HPLC-grade dichloromethane. Then, 100 µL aliquots were transferred into sterile GC-MS vials. The solvent was evaporated under nitrogen stream, and the residue was reconstituted in 100 µL of dichloromethane prior to injection. The ionization energy was 70 eV. The injector and detector temperatures were set at 260 °C and 290 °C, respectively. The temperature program began with 40 °C (1 min), increased to 100 °C at 60 °C/min, then to 290 °C at 4 °C/min, holding for 2 min. The interface temperature was 300 °C. Helium was used as the carrier gas (1 mL/min), and 2 µL of the sample was injected in splitless mode. Mass spectrum data were obtained using Scan analysis mode (40–450 *m*/*z*) scan range, and compound identification was based on spectral matching with literature [32]. Relative abundances were calculated from chromatographic peak areas using OpenChrom^®^ software version 1.5.11.

Complementary chemical analyzes were performed by ^1^H NMR were using Varian^®^ VNMRS spectrometers (500 MHz for 1H). CDCl_3_ was used as a solvent for the samples and Tetramethylsilane (TMS) was used as internal reference. Chemical shifts (δ) were expressed in parts per million (ppm) and coupling constants in Hertz (Hz). The relative areas of the signals were obtained by electronic integration, and the calibration of the spectra was obtained from the TMS signal. Spectra processing was performed using MestReNova software version 6.0.2.IR spectra.

Moreover, the Fourier-Transform Infrared Spectroscopy (FT-IR) spectra of the solid samples, prepared as KBr pellets, were recorded using an FTIR-IRTracer 100 Shimadzu spectrometer. Data acquisition was performed with a DTGS detector and a KBr beam-splitter. The spectra were collected within the 4000–400 cm^−1^ range at a resolution of 4 cm^−1^. Additional characterization of water-soluble fraction (CCFPOL) was performed for the quantification of total proteins and phenolic compounds, following the methodology described by Santos et al. [44]. The presence of proteins on crude polysaccharide fraction was estimated by the Bradford assay, using bovine serum albumin as a standard [45]. Additionally, the total phenolic content was evaluated by the Folin–Ciocalteu method [46].

### 2.5. Bio-Screening Assays

The extracts (ECCD and ECCH) and fractions from *C. cervicornis*, were subjected to in vitro biological assays, to evaluate their cytotoxicity, neuroprotective effects against 6-OHDA-induced neurotoxicity and their mechanism of action through quantification of hydrogen peroxide, alterations in mitochondrial membrane potential and intracellular ROS production.

#### 2.5.1. Cell Culture Maintenance

Murine fibroblast cell line (3T3) and human neuroblastoma cell lines (SH-SY5Y) ACC 173 and ACC 209) were obtained from the German Collection of Microorganisms and Cell Cultures GmbH (DSMZ) Bank. Cells were maintained at 37 °C and 5% CO_2_ with DMEM: F12 medium supplemented with 10% (*v*/*v*) fetal bovine serum (FBS) and 1% antibiotic/antimycotic solution (Amphotericin B, Penicillin and Streptomycin) (Biowest, Nuaillé, France). For subculturing, cells were dissociated with trypsin-EDTA and split at a 1:3 ratio. A density of 5 × 10^4^ cells/well was seeded in 96-well plates for cell viability and neuroprotective assays.

#### 2.5.2. Cytotoxic and Neuroprotective Effects Against 6-OHDA-Induced Neurotoxicity

The cytotoxicity of seaweed fractions in both cell lines and neuroprotection assays were estimated using the 3-[4, 5-dimethylthiazol-2-yl]-2, 5-diphenyltetrazolium bromide (MTT) (VWR, Solon, OH, USA) method, as described by Silva et al. [20,21]. A preliminary cytotoxicity assay using 3T3 (Supplementary data) and SH-SY5Y cells were used to determine the non-toxic concentrations of the extracts and fractions before proceeding to neuroprotection assays. The SH-SY5Y cells were exposed 24 h to 6-OHDA (100 µM) in the absence (Vehicle) or presence of extracts and fractions at concentrations of 0.1–100 µg/mL. Then, 100 µL MTT (1.2 mM), previously prepared in medium without FBS, were added to wells and cells were incubated for 1 h at 37 °C. After this time, MTT was removed and DMSO (100 µL) was added to dissolve the formazan crystals. The resulting absorbance was read in a microplate reader (Biotek Epoch/2, Winooski, VT, USA) at 570 nm. The results were expressed in percentage of control.

#### 2.5.3. Quantification of Hydrogen Peroxide (H_2_O_2_)

This assay was performed using the Amplex Red Hydrogen Peroxide/Peroxidase Assay kit (Life Technologies, Camarillo, CA, USA), as previously described by Silva et al. [20]. The H_2_O_2_ production was quantified in SH-SY5Y cells after 6 h of treatment with 6-OHDA (100 µM) in the absence or presence of samples from *C. cervicornis*. The variation of H_2_O_2_ production was accompanied in real-time for 30 min at room temperature. Results were expressed in percentage of control.

#### 2.5.4. Mitochondrial Membrane Potential (MMP)

Alterations in mitochondrial membrane potential was determined using the fluorescent probe, JC-1 (Molecular Probes, Eugene, OR, USA), according to the method described by Silva et al. [20]. The SH-SY5Y cells were treated with 6-OHDA (100 µM) for 6 h, in the absence or presence of samples from *C. cervicornis*. Results were expressed as the ratio of the monomers/aggregates of JC-1 in percentage of control.

#### 2.5.5. Intracellular ROS Production

The detection of ROS generated in injured cells was evaluated using the 5(6)-carboxy-2′,7′-dichlorofluorescein diacetate (carboxy-H2DCFDA) reagent (Invitrogen, Bleiswijk, The Netherlands) as described in Silva et al. [20]. The SH-SY5Y cells were treated with 6-OHDA (100 µM) for 6 h, in the absence or presence of samples from *C. cervicornis*. After incubation, cells were washed with ice-cold Hanks’ balanced salt solution buffer (Hbss). Then, the carboxyl-H_2_DCFDA (100 µL, 20 µM) reagent was added and incubated for 1 h at 37 °C. Finally, the fluorescence intensity was measured at wavelengths of λ527 nm (excitation) and λ590 nm (emission), and ROS levels were presented in percentage of control (non-treated cells).

### 2.6. Data and Statistical Analysis

The results are presented as mean plus standard error of the mean (SEM). A paired Student’s *t*-test was used to compare two groups. For comparisons involving three or more groups, a one-way analysis of variance (ANOVA) followed by Dunnett’s multiple comparison test to assess significant differences relative to the control treatment were performed. All data were checked for normality using the Shapiro–Wilk test. Comparisons concerning variables, which did not meet variance or distributional assumptions, were carried out with the Kruskal–Wallis non-parametric test. Differences were considered significant at the level of 0.05 (*p* < 0.05). Additionally, the IC_50_ values were determined using the software GraphPad v9.1 using the equation y = 100/(1 + 10 (X − log IC_50_)). All results were obtained from three or four independent experiments.

## 3. Results and Discussion

### 3.1. Chemical Characterization of Extracts and Fractions from C. cervicornis

The brown seaweed *C. cervicornis* contains mostly unique cyclic diterpenoids that are associated with a plenty of biological activities [35,40,41]. This study presents a targeted methodology for the fractionation and characterization of extracts and their respective fractions (lipophilic and water-soluble) from *C. cervicornis*.

The lipophilic extraction was performed using dichloromethane, while the more polar extract was obtained with a mixture of ethanol and water (70:30, *v*/*v*). Additionally, a water-soluble fraction rich in polysaccharides (CCFPOL) was obtained through ethanol precipitation of ECCH, followed by successive washes with ethanol and acetone, and vacuum filtration. The yields and FT-IR data for ECCD, ECCH, and CCFPOL are presented in Table 1. The yield of the dichloromethane extract was 10.48%, while the 70% ethanolic extract exhibited a yield of 9.39%. Ethanol precipitates proteins and polysaccharides, leading to a lower yield of hydroethanolic extracts. However, the yield of CCFPOL was 2.03%.

It is important to highlight that most of the literature reports the identification and isolation of diterpenes from *C. cervicornis* using dichloromethane [37,38,39,40,47,48,49,50,51]. In contrast, the extraction of polysaccharides from this seaweed was described by Camara et al. [52] using an enzymatic extraction followed by precipitation with acetone.

**Figure 3:**
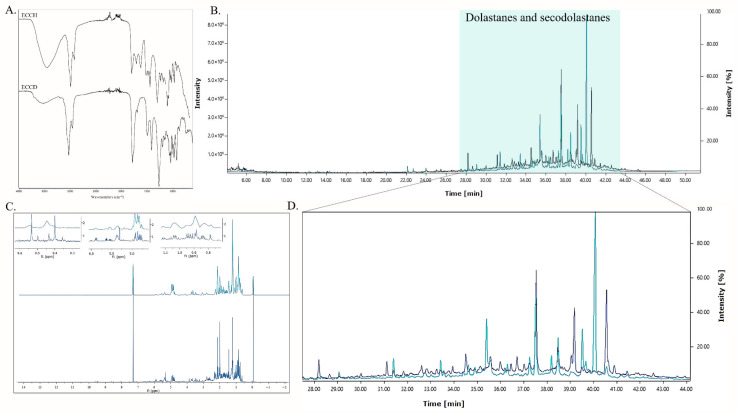
(**A**) FT-IR spectra of the dichloromethane extract (ECCD) and hydroethanolic extract (ECCH) from the brown seaweed *Canistrocarpus cervicornis*. (**B**) GC-MS spectra of ECCD (dark blue) and ECCH (light blue). (**C**) ^1^H NMR spectra of ECCD and ECCH. (**D**) Amplification of the GC-MS chromatographic region (28–44 min) of ECCD (dark blue) and ECCH (light blue).

Based on the wavelength and intensity of the absorption bands of different molecular groups, FT-IR spectroscopy can reveal the presence of different chemical components (Figure 3A). The ECCD and ECCH extracts of *C. cervicornis* showed an absorption band at 3392–3401 cm^−1^ (OH stretch alcohol). It is noteworthy that the ECCH extract presented greater intensity of this signal, attributed to a higher concentration of hydroxylated polar compounds, as commonly observed in hydroethanolic extracts. All extracts of *C. cervicornis* showed a strong peak between 2955 and 2926 cm^−1^ (C-H stretching vibrations). The band range of 1732 cm^−1^ suggests the presence of C = O characteristic of esters or ketone groups. The FT-IR spectra revealed the presence of various chemical groups associated with the bioactive molecules present in *C. cervicornis*.

The FT-IR spectra of the water-soluble fraction (CCFPOL) showed the typical absorption bands of polysaccharides and other hydrophilic constituents (Supplementary data, Figure S1). The bands around 3336 and 2924 cm^−1^ were assigned to the deformation of O-H and C-H stretching vibrations, respectively. The absorption peaks at 1612 and 1412 cm^−1^ indicated the presence of carbonyl groups. The fucoidan characteristic absorption bands at 1240~1260 cm^−1^ (S = O stretching vibration) suggests the presence of sulfate groups [53,54].

Chemical methods including the quantification of proteins and phenolics by, respectively, the Bradford and Folin methodologies were applied to the additional characterization of this fraction (Supplementary data, Table S5). No detectable levels of phenolic compounds or proteins were observed. These results suggest that CCFPOL has minimal contamination by phenolics or proteinaceous material; however, other additional techniques should be performed to expand the characterization of the fraction, which include X-ray diffraction, analysis of monosaccharide composition by high-performance chromatography techniques, molecular weight analysis, among others [55,56,57].

It should be noted that obtaining polysaccharides from seaweeds usually involves steps such as aqueous extraction, enzymatic extraction or acid hydrolysis, precipitation with organic solvents and, often, dialysis to remove salts and other low molecular weight impurities [58]. Previous studies with seaweeds of the Dictyotaceae family have performed the extraction of these polysaccharides without the dialysis step [54,59,60]. Cui et al. [60] performed the extraction of polysaccharides from the seaweed *Dictyopteris divaricata* with deionized water followed by vacuum extraction, precipitation, and washing with ethanol. The precipitate showed characteristic signs of sulfated polysaccharides. Costa et al. [54] described the extraction of sulfated polysaccharides from 11 species of seaweed, including species from the family Dictyotaceae, *D. menstrualis*, *D. mertensii* and *C. cervicornis*, using enzymatic hydrolysis followed by precipitation with acetone. Although dialysis is a common step in the purification of polysaccharides extracted from algae, there are cases in the literature where this step was not performed. However, this process can influence the purity of the polysaccharides, which may be important for specific applications [58].

**Figure 4:**
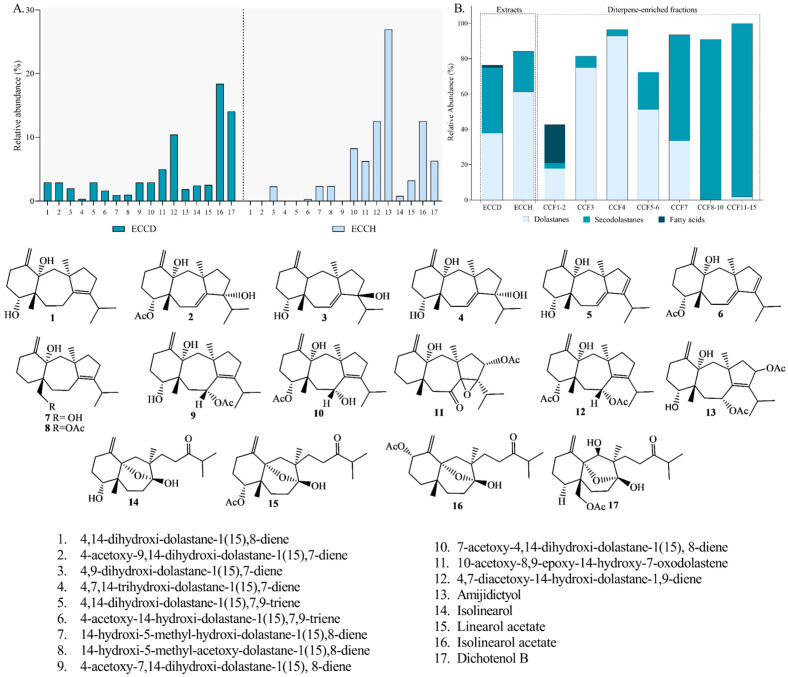
Lipophilic profile of extracts (dichloromethanic (ECCD) and hydroethanolic (ECCH)) and fractions (CCF1–2-CCF11–15) from *Canistrocarpus cervicornis* identified by GC-MS analysis. (**A**) Comparison of the relative abundance of major diterpenes in ECCD and ECCH. (**B**) Comparison of the relative abundance of identified compound groups (dolastanes, secodolastanes, and fatty acids).

The lipophilic profile of *C. cervicornis* was analyzed using GC-MS. In this study, 17 diterpenoids were putatively detected in the extracts through GC-MS analysis (Figure 4A, Table S1). The most abundant compounds in ECCD were identified as the secodolastane isolinearol acetate (C_22_H_34_O_5_), dichotenol B (C_22_H_34_O_6_), and the dolastane 4,7-diacetoxy-14-hydroxy-dolastane-1,9-diene (C_24_H_36_O_5_), constituting 18.39%, 14.08%, and 10.43% of the total extract, respectively. On the other hand, the most abundant diterpenoids identified in ECCH were amijidictyol (C_24_H_36_O_6_; 26.96%), followed by isolinearol acetate (12.53%) and 4,7-diacetoxy-14-hydroxy-dolastane-1,9-diene (12.52%). The literature highlights that this species predominantly produces diterpenes, and the proportions of the identified compounds are consistent with previous studies [38,40,48,49,50,61,62].

The presence of diterpenes was confirmed by ^1^H NMR analysis. Based on literature data, singlet signals associated with methylene hydrogens of C-16 and C-20 (δ_H_ 0.60–1.00 and 1.20–1.50), along with the doublet signals of methyl groups C-19 and C-18 (δ_H_ 0.83–0.88 and 1.03–1.05) and an exocyclic doublet at δ_H_ 4.60–5.10, suggest the presence of dolastane and secodolastane skeleton compounds [38,48,50]. Notably, the methyl doublet signals could also indicate the presence of an isopropyl group, which warrants further structural evaluation.

Most studies on the isolation and identification of diterpenes from *C. cervicornis* have been conducted using organic extracts, particularly dichloromethane extracts. Obando et al. [63] identified diterpenes with dolastane and secodolastane skeletons in aqueous extracts of this alga. The identification of these metabolites in a polar extract using less toxic solvents, such as water and a hydroethanolic mixture (70:30, *v*/*v*), paves the way for greener and lower cost to obtain these compounds.

**Figure 5:**
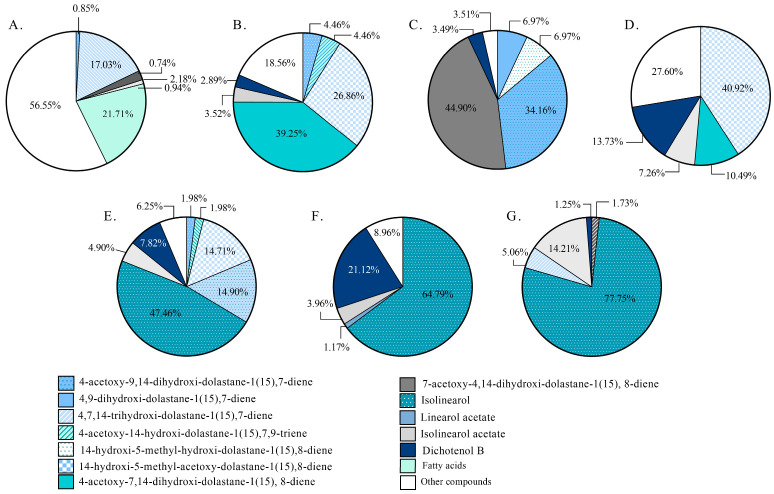
Comparison of the relative abundance of major diterpenes in diterpenoid-rich-fractions from *Canistrocarpus cervicornis* identified by GC-MS analysis. The charts represent a specific fraction: CCF1–2 (**A**); CCF3 (**B**); CCF4 (**C**); CCF5–6 (**D**); CCF7 (**E**); CCF8–10 (**F**); and CCF11–15 (**G**).

Regarding the diterpene-enriched fractions, the presence of 12 diterpenes with dolastane and secodolastane skeletons was identified (Figure 5A–G). It was observed that the CCF1–2 fraction exhibited a higher presence of fatty acids compared to the monitored diterpenes (21.70%). The CCF3, CCF4, and CCF5–6 fractions showed a higher proportion of dolastane-type diterpenoids (75.04%, 92.99%, and 51.40%, respectively) (Figure 4B). In contrast, the CCF7, CCF8–10, and CCF11–15 fractions displayed higher proportions of secodolastane-type diterpenes (60.07%, 91.03%, and 98.27%, respectively), with isolinearol and isolinearol acetate being the major metabolites. The mass spectrum of isolinearol acetate exhibited a molecular ion at *m/z* 378, along with fragment ions at *m/z* 319 [M^+^-OAc], 302 [M^+^-OAc-H_2_O], and 43 [CH_3_CO]^+^, among others (Table S1). The isolation and elucidation of the compounds are currently in progress for further subjection to biological assays.

### 3.2. Neuroprotective Potential of Extracts and Fractions from C. cervicornis on In Vitro Cellular Models

The 3T3 fibroblast line, a non-tumoral cell model, was used solely for preliminary cytotoxicity screening for early safety evaluation [64,65,66,67]. This model enabled a rapid and cost-effective assessment of extract and fraction toxicity, supporting the selection of safe concentration ranges prior to the neurotoxicity assays in SH-SY5Y cells. As the 3T3 model is not directly related to neuroprotective mechanisms, its results are presented in the Supplementary Material (Figure S13). Similar approaches have been reported in pharmacological safety assessments before neuronal study [65,67]. Li et al. [65] investigated the cytotoxic activity of the synthetic compounds in human keratinocytes (HaCaT) and 3T3 in a study primarily aimed at evaluating their neuroprotective potential against β-amyloid_25–35_-induced neurotoxicity in SH-SY5Y cells, emphasizing that assessing compound toxicity profiles is a crucial step in the drug research and development process.

**Figure 6:**
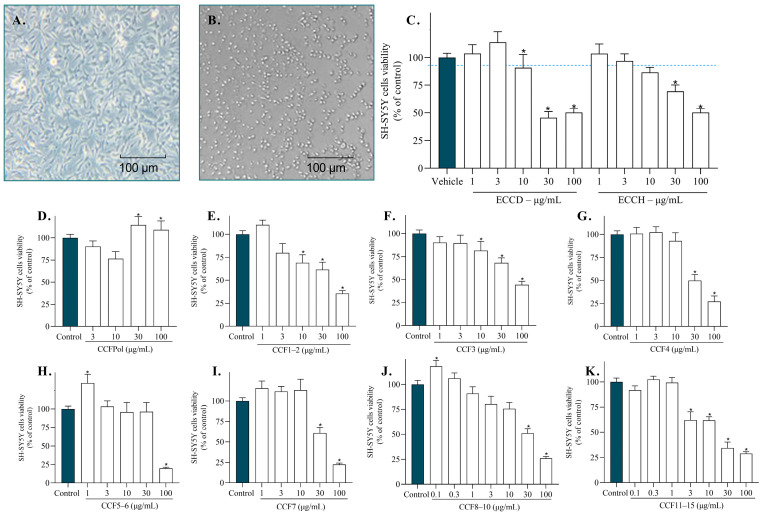
Cytotoxicity of *Canistrocarpus cervicornis* extracts and fractions (0.1–100 µg/mL; 24 h) on SH-SY5Y cells. Representative photograph of SH-SY5Y cells treated with the vehicle DMSO (**A**) and the cell death control saponin (0.4 mg/mL; 24 h) (**B**). Cytotoxicity of dichloromethane extract (ECCD) and hydroethanolic extract (ECCH) (**C**); CCFPOL (**D**); CCF1–2 (**E**); CCF3 (**F**); CCF4 (**G**); CCF5–6 (**H**); CCF7 (**I**); CCF8–10 (**J**); CCF11–15 (**K**). The values in each column represent the mean ± standard error of the mean (SEM) of 3 or 4 independent experiments. The symbol (*) represents significant differences (*p* < 0.05; Paired Student’s *t*-test). Vehicle (DMSO (1%) or H_2_O) was considered as 100% viable cells.

Cytotoxic effects of extracts and fractions from *C. cervicornis* were assessed by the MTT assay after 24 h of incubation in SH-SY5Y cells. Figure 6 shows the results obtained for tested extracts and fractions in cellular viability. At a concentration of 100 µg/mL, the ECCH extract exhibited a cell viability of 47.75 ± 9.65%, whereas ECCD showed a significantly reduced viability of 34.34 ± 8.59%. These results highlight the influence of extract composition on cell viability, largely attributed to the use of organic solvents such as dichloromethane in the preparation of ECCD. This solvent is known to promote the extraction of less polar compounds, which often exhibit higher toxicity [68].

At 30 µg/mL, the highest concentration tested for CCF1–2, CCF3, CCF4, CCF7, CCF8–10, and CCF11–15, cell viability ranged from 49.18% to 97.43% (Figure 6C–K). Meanwhile, the polysaccharide fraction (CCFPOL) did not exhibit toxicity at the maximum tested concentration (100 µg/mL). In the SH-SY5Y cell model, IC_50_ values revealed variations in cytotoxicity among the tested extracts and fractions. Among the extracts, ECCD presented an IC_50_ value of 29.55 µg/mL, indicating high toxicity. The extract ECCH showed moderate toxicity (IC_50_ 65.31 µg/mL). Regarding the fractions, CCF1–2 exhibited high toxicity with an IC_50_ value of 29.44 µg/mL. In contrast, the fraction CCF3 displayed lower toxicity (IC_50_ > 100 µg/mL). Fractions CCF4 and CCF5–6 showed an IC_50_ value of 48.04 µg/mL and 57.04 µg/mL, respectively. The fraction CCF7 exhibited an IC_50_ value of 40.17 µg/mL, CCF8–10 presented a value of 19.53 µg/mL and CCF11–15 displayed the lowest IC_50_ value (14.27 µg/mL), indicating higher toxicity.

**Figure 7:**
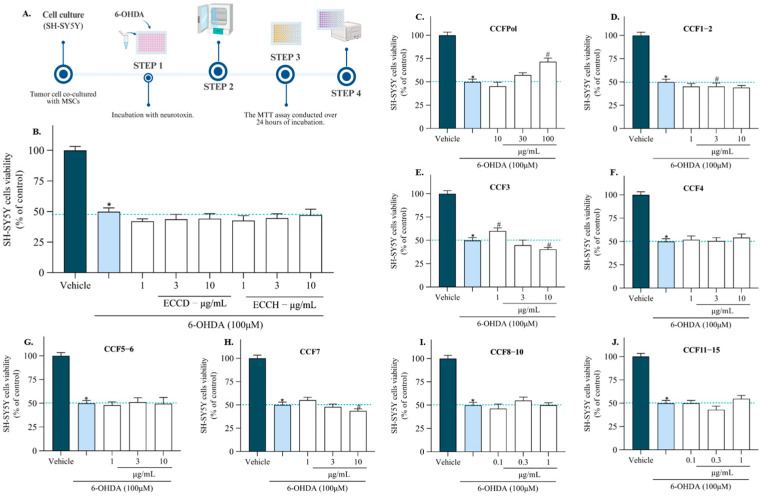
Neuroprotective potential against 6-OHDA-induced neurotoxicity of *Canistrocarpus cervicornis* extracts and fractions (0.1–100 ug/mL; 24 h) on SH-SY5Y cells. (**A**) Graphical representation of workflow: SH-SY5Y cells exposed to the neurotoxin 6-hydroxydopamine (6-OHDA), followed by a 24 h incubation and cell viability assessment using the MTT assay. Cytotoxicity of the dichloromethane (ECCD) and hydroethanolic extracts (ECCH) on SH-SY5Y cells (**B**); CCFPOL (**C**); CCF1–2 (**D**); CCF3 (**E**); CCF4 (**F**); CCF5–6 (**G**); CCF7 (**H**); CCF8–10 (**I**); CCF11–15 (**J**). The values in each column represent the mean ± standard error of the mean (SEM) of 3 or 4 independent experiments. The symbol (*) represents significant differences compared to the vehicle and the symbol (#) represents significant differences compared to 6-OHDA (*p* < 0.05). Vehicle control (untreated cells) was considered as 100% viable cells. Dark blue bars (untreated cells; vehicle); light blue bars (cells exposed to 6-OHDA).

The neuroprotective potential of extracts and fractions from *C. cervicornis* (0.1–100 µg/mL; 24 h) were evaluated in the presence of the 6-OHDA neurotoxin (Figure 7B–J) at sub-toxic concentrations. The exposure of SH-SY5Y cells to 6-OHDA (100 µM) for 24 h reduced the cell viability by about 50.70 ± 2.71% of viable cells. The ECCD (41.63–50.74%) and ECCH (36.98–52.08%) extracts did not revert the neurotoxicity induced by 6-OHDA treatment on SH-SY5Y. On the other hand, the CCFPOL (100 µg/mL; Figure 6C) and CCF3 (1 µg/mL; Figure 6E) showed a significant increase in cell viability when compared with 6-OHDA treatment exhibiting a value of 68.43 ± 4.60% and 60.61 ± 0.80% of viable cells, respectively. This corresponds to an increase of 17.69% and 9.87% in cell viability compared to neurotoxin-treated cells. The other fractions did not show a significant ability to neutralize 6-OHDA-induced toxicity. Although only CCF3 showed ability to recover the neurotoxicity induced by 6-OHDA, other non-active fractions also presented diterpenoid-rich profiles. However, differences in the relative abundance of major and minor compounds among these fractions likely influence their biological behavior, indicating that subtle variations in composition can impact the observed activity.

The neurotoxin 6-OHDA is widely used to mimic the condition of PD in an in vitro and in vivo models, triggering intracellular events that lead to the death of dopaminergic neurons [66,67,69]. Its effects are primarily related to oxidative stress, as once accumulated in the cytosol, it can undergo auto-oxidation, leading to a high rate of free radical generation [70,71]. This neurotoxin has also been applied in studies on other neurological disorders, including ASD and attention deficit hyperactivity disorder (ADHD) [72,73]. Alhamami et al. [72] investigated the effect of inhibiting A2 noradrenergic neurons in the hindbrain with 6-OHDA in an autistic animal model. The authors reported that the 6-OHDA can activate neuroinflammatory pathways in the prefrontal cortex and hippocampus of autistic animal models. Similar mechanisms appear to be involved in the etiology of PD, AD, and ASD [8,18,23,70]. Some of the oxidative damage includes lipid peroxidation, protein degradation, mitochondrial dysfunction, and cell death [70].

### 3.3. Cellular Mechanisms Associated with the Neuroprotective Potential of C. cervicornis

**Figure 8:**
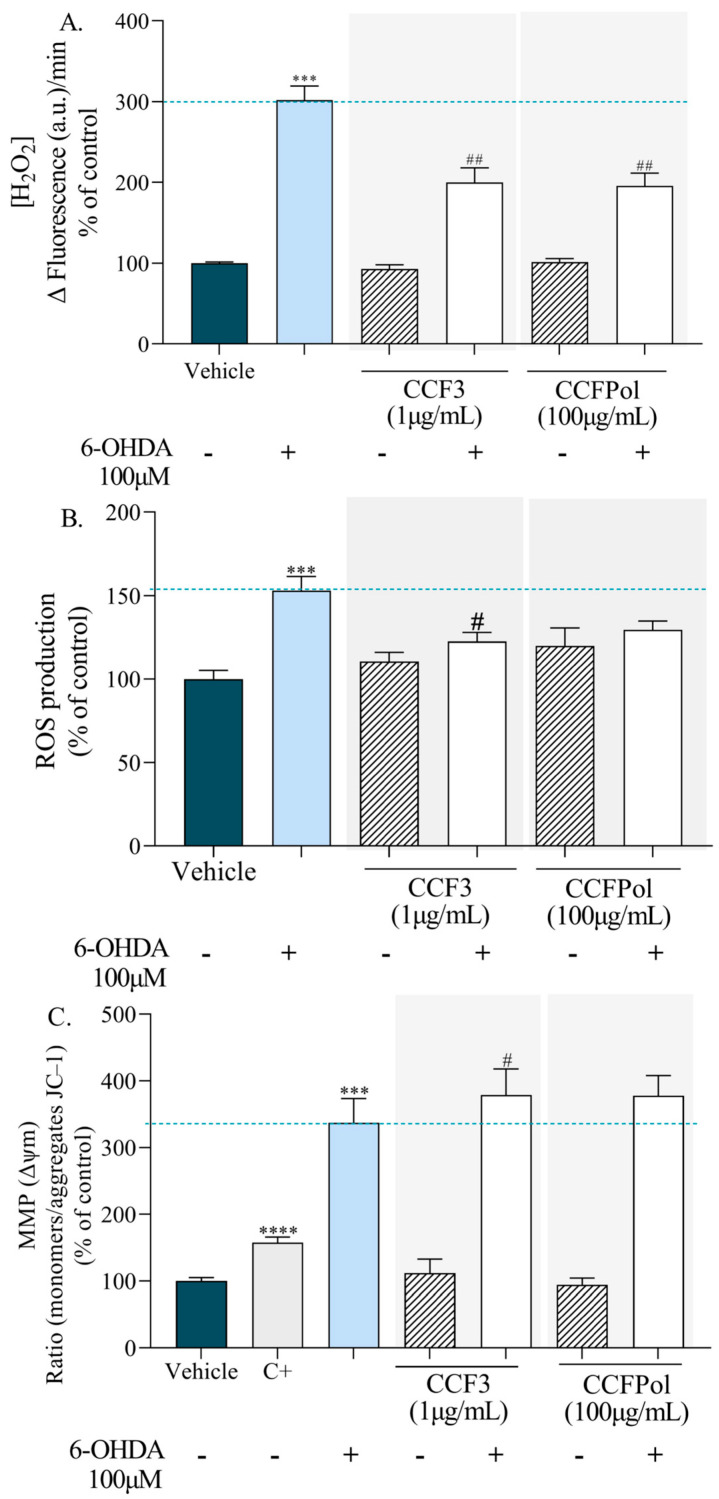
Hydrogen peroxide (H_2_O_2_), reactive oxygen species (ROS) and alterations in mitochondrial membrane potential (MMP) levels in SH-SY5Y cells treated with fractions from *Canistrocarpus cervicornis*. (**A**) Levels of H_2_O_2_ produced by SH-SY5Y cells after 6 h of treatment with 6-OHDA (100 µM) in the presence/absence of seaweed fractions (1–100 µg/mL, 6 h). H_2_O_2_ quantification was performed fluorometrically using the Amplex Red Hydrogen Peroxide/Peroxidase Assay kit. (**B**) ROS production of SH-SY5Y cells treated with 6-OHDA (100 µM) in the presence/absence of fractions (1 or 100 µg/mL, 6 h). (**C**) Changes in mitochondrial membrane potential (MMP) of SH-SY5Y cells following treatment with 6-OHDA (100 µM) in the presence/absence of fractions (1 or 100 µg/mL, 6 h). MMP was assessed by the JC-1 monomer/aggregate ratio. FCCP (2.5 µM) and oligomycin A (1 mg/mL) conjugate solution was used as positive control (C+). The symbol (+) indicates the presence, while (–) indicates the absence of 6-OHDA. Data is presented as mean ± SEM from three to four independent experiments. Statistical significance was determined by ANOVA followed by Dunnett’s test: *** *p* < 0.001, **** *p* < 0.0001 vs. vehicle; # *p* < 0.05, ## *p* < 0.01 vs. 6-OHDA. Dark blue bars (untreated cells; vehicle); light blue bars (cells exposed to 6-OHDA); gray bars (positive control; C+); hatched bars (cells treated with the fractions in the absence of 6-OHDA); white bars (co-treated with the fractions and 6-OHDA).

The neuroprotective potential of *C. cervicornis* on the viability of SH-SY5Y cells was assessed through in vitro assays, including changes in MMP, H_2_O_2_ production, and ROS generation. These assays were conducted on cells exposed to the neurotoxin 6-OHDA, either in the absence or presence of *C. cervicornis* bioactive diterpene- and polysaccharide-enriched fractions CCF3 and CCFPOL, respectively, following a 6 h incubation. Figure 8 shows the results of the changes in MMP, H_2_O_2_ and ROS production in SH-SY5Y cells. The results were presented as a percentage of control (cells without treatment).

The treatment with 6-OHDA (100 µM, 6 h) led to a marked increase in H_2_O_2_ production (302.2 ± 17.07% of vehicle, *p* < 0.001) (Figure 8A). When treated with CCF3 (1 µg/mL), H_2_O_2_ levels were reduced to 200.0 ± 18.19%, and with CCFPOL (100 µg/mL) decreased them to 195.5 ± 16.13%. It is noteworthy that the fractions did not increase basal H_2_O_2_ levels when administered without the neurotoxin (93.14 ± 5.15% for CCF3 and 101.4 ± 4.46% for CCFPOL).

In addition, the exposure of SH-SY5Y cells to 6-OHDA increased intracellular ROS levels to 153.0 ± 8.43% relative to vehicle (Figure 8B). The treatment with *Canistrocarpus cervicornis* fractions attenuated this effect, with CCF3 (1 µg/mL) reducing ROS levels to 122.6 ± 5.35% (*p* < 0.05) and CCFPol (100 µg/mL) to 129.6 ± 5.18 when compared with cells exposed to neurotoxin. These findings highlight a protective role of the fractions against 6-OHDA–induced oxidative stress.

The literature reports that 1% to 2% of the electrons produced by the mitochondria can react with molecular oxygen, sequentially forming ROS such as superoxide and hydrogen peroxide, that can activate apoptotic pathways, leading to cell death [20]. Given this, the reduction in the neurotoxic effect of 6-OHDA, as evidenced by the CCFPOL and CCF3 fractions, seems to be associated with different interrelated mechanisms, particularly a decrease in ROS and H_2_O_2_ production. These fractions exhibit distinct chemical compositions, CCF3 being rich in diterpenoids and CCFPOL rich in polysaccharides, suggesting that different classes of metabolites may contribute to the observed antioxidant and protective actions. Studies on brown seaweeds have reported antioxidant potential, including polysaccharide-rich extracts from *Canistrocarpus* species [53] and diterpenoid fractions from *Bifurcaria* and *Dictyota* genera [20,33]. These findings not only support the results obtained in the present study but also highlight the need for further evaluation and exploration of native brown algal substances and fractions exhibiting similar bioactive potential.

The treatment with the CCFPOL and CCF3 fractions without 6-OHDA did not change the mitochondrial membrane potential compared with the vehicle (Figure 8C). The exposure of SH-SY5Y cells to 6-OHDA (100 µM, 6 h) induced a strong depolarization of the MMP when compared with the vehicle (*p* < 0.001). To support the interpretation of MMP alterations, a positive control composed of carbonyl cyanide-4-(trifluoromethoxy)phenylhydrazone (FCCP), and oligomycin A, which are known to disrupt mitochondrial function was used [74]. This combination induced a shift toward the monomeric form of JC-1, consistent with mitochondrial depolarization. This control served to corroborate the interpretation of JC-1 ratio changes observed in the experimental treatments. The rise in the JC-1 monomer/aggregate ratio observed in CCF3-treated cells reflects mitochondrial depolarization, indicating increased mitochondrial dysfunction.

These results align with those obtained by Silva et al. [20], who demonstrated that SH-SY5Y cells exposed to the neurotoxic effects of 6-OHDA and treated with a diterpene-fraction from the brown seaweed *Bifurcaria bifurcata* showed prevention of mitochondrial membrane potential changes, a decrease in H_2_O_2_ production, and increased cell viability. It is important to highlight that this study explores an enriched and complex fraction, in which the observed effects likely reflect the combined action of multiple bioactive metabolites [75,76]. This inherent variability in natural fractions highlights the dynamic interplay among metabolites, and provides a preliminary data for future studies aimed at elucidating specific mechanisms of action of the compounds.

A previous study on polysaccharides from marine organisms also reported a reduction in ROS levels and an increase in cell viability [77]. Bastos et al. [67] reported that the polysaccharide ɩ-carrageenan from the red algae *Solieria filiformis* has a superior effect on mitochondrial protection and maintained H_2_O_2_ generation at basal levels. In this study, we observed the neuroprotective potential of the polysaccharide fraction CCFPOL. However, this effect should be further evaluated in specific vivo models, considering the low cellular permeability and the limited ability of polysaccharides to cross the blood–brain barrier (BBB), is necessary for future applications [78,79]. A promising strategy to overcome this limitation is the conjugation of these molecules with nanoparticles, leveraging nanotechnology to enhance their transport across the BBB [78].

Other seaweed-derived compounds have also been reported for their neuroprotective properties. In particular, fucoidans and phlorotannins from brown algae have demonstrated antioxidant, anti-inflammatory, and cytoprotective effects in neuronal cell models, suggesting that multiple classes of algal metabolites can act as neuroprotective agents [78,80]. Sulfated polysaccharides from the brown seaweed *Saccharina japonica* (Laminariaceae) have demonstrated protective effects in SH-SY5Y cells against H_2_O_2_-induced cytotoxicity, mediated by activation of the PI3K/Akt signaling pathway [81]. Similarly, fucoidan from *Fucus vesiculosus* attenuated 6-OHDA-induced neurotoxicity in SH-SY5Y cells by exerting anti-oxidative and anti-apoptotic effects; notably, treatment with fucoidan (40 µg/mL) significantly inhibited the loss of MMP by 16.7% compared to 6-OHDA-treated cells [82]. Additionally, Dieckol, a phlorotannin isolated from the brown alga *Ecklonia cava*, decreased ROS generation under the rotenone-induced oxidative stress in SH-SY5Y cells [24]. Another study related that a phlorotannin isolated from the brown algal species, *Padina tetrastromatica* (Dictyotaceae), further demonstrated significant free-radical scavenging activity in several cell lines, including the SH-SY5Y cell line [83].

Regarding the neuroprotective potential of algae from the Dictyotaceae family, several diterpenes produced by algae of this family have demonstrated neuroprotective and anti-inflammatory effects. Recently, the diterpene 18-acetoxy-6,7-epoxy-4-hydroxydictyo-19-al obtained from the species *Dictyota coriacea* was related to the activation of the Nrf2/ARE signaling pathway, demonstrating neuroprotective effects in an in vitro and in vivo models [84]. The prenylated guaian skeleton compound, dictyol C, isolated from the species *Dictyota* sp. collected in China, showed a potent antioxidant effect against H_2_O_2_-induced oxidative damage in neuronal-like PC12 cells at a low concentration of 2 μM, suggesting neuroprotective potential [85].

Diterpenoids from the species *D. menstrualis* [4,5,6,7,8,9,10] were investigated for their anti-inflammatory action, demonstrating inhibitory activity against nitric oxide (NO) production on the RAW 264.7 cell line stimulated by LPS (IC_50_ 0.12–0.23 mM) [86]. Furthermore, the *n*-hexane: ethyl acetate (85:15; *v*/*v*) and ethyl acetate fractions of *C. cervicornis* showed anti-inflammatory effects related to the inhibition of nitric oxide and the cytokines IL-6 and IL-10 in assays with macrophages isolated from the peritoneal cavity of mice in the presence or absence of lipopolysaccharide (LPS) at 1 µg/mL [87].

A recent study using diterpene-enriched fractions from *D. menstrualis* demonstrated anti-inflammatory effects in a zebrafish model [88]. Fractions composed mainly of xeniane-type diterpenes (group III) and Dictyol C (group I) (DEF-1), as well as DEF-3, also rich in xeniane-type diterpenes, effectively inhibited neutrophil recruitment to the site of injury during both early and late stages of inflammation. This fraction-based approach offers both biological insights and a more sustainable research pathway, serving as an initial bioguided step before full compound isolation while enabling the exploration of potential synergistic interactions that may enhance biological effects [89]. In this context, the use of extracts with different polarities and subsequent fractionation constitutes a metabolomic and bioassay-guided strategy that connects chemical diversity to biological activity, supporting the identification of bioactive fractions and guiding future purification and mechanistic studies.

It is important to emphasize that further efforts should be directed toward the isolation, purification, and characterization of the bioactive metabolites of *C. cervicornis*, to deepen our understanding of the neuroprotective potential of the compounds from this seaweed. Furthermore, future work will focus on applying analytical techniques such as liquid chromatography–mass spectrometry (LC–MS) and quantitative NMR to determine the purity of components in both lipophilic and hydrophilic fractions, thereby refining chemical characterization and supporting structure–activity analyses. Future studies could also involve pre-treatment approaches with isolated compounds, to further elucidate the mechanisms underlying the recovery from 6-OHDA-induced neurotoxicity.

Bioinformatics strategies can be employed to optimize compound screening and study design, particularly through target-ligand interaction analyses such as molecular docking [90,91]. These tools may assist in elucidating potential mechanisms of action and prioritizing the most promising compounds, being aligned with more sustainable practices and optimization of solvents and reagents use [91].

Furthermore, in silico predictive tools can be applied to assess the ability of metabolites to cross BBB, and obtain further information on metabolite absorption and interaction, such as ADMETlab and SwissADME [92]. Finally, the advancement of in vitro studies using more complex and specific cellular models, including differentiated cells [23,92], alongside the investigation of additional mechanisms of action, mainly related to anti-neuroinflammatory activity, represent an avenue for expanding the neuroprotective potential knowledge of *C. cervicornis* in the context of neurological disorders.

## 4. Conclusions

This study presents the chemical characterization of the dichloromethane and hydroethanolic extracts, as well as fractions from the brown alga *Canistrocarpus cervicornis*. A total of 17 diterpenes with dolastane and secodolastane skeletons were putatively identified by spectroscopic analysis and comparison with previously reported data. In addition, a preliminary characterization of the polysaccharide fraction (CCFPOL) of this alga is provided. The extracts and fractions were evaluated for their neuroprotective activity in the human neuroblastoma cell model SH-SY5Y treated with the neurotoxin 6-OHDA. This is the first report evaluating the neuroprotective potential of *C. cervicornis* for applications in neurological disorders.

Our results indicate that the CCFPOL and CCF3 fractions exhibited ability to recover the neurotoxicity mediated by the treatment with 6-OHDA on SH-SY5Y cells. These fractions protected against neuronal death by reducing H_2_O_2_ and ROS production. Future studies should prioritize the isolation of the *C. cervicornis* compounds and the elucidation of the specific mechanisms underlying their contributions to the observed bioactivities. The in vitro assays conducted in this study highlight the potential of seaweed-derived compounds as natural therapeutics to prevent and counteract oxidative stress-induced neurodegeneration.

## Figures and Tables

**Table 1:** Yield and FT-IR data of the dichloromethane extract (ECCD), hydroethanolic extract (ECCH), and polysaccharide-rich fraction (CCFPOL) from *Canistrocarpus cervicornis*.

Sample	Yield (% of Dry Weight)	IR in KBrv_max_/cm^−1^
ECCD	10.48	3401; 2926; 2855; 1732; 1643; 1454; 1374; 1233; 1166; 1134; 1033; 1014; 978; 953; 901; 849; 732
ECCH	9.39	3392; 2924; 2854; 1732; 1642; 1455; 1375; 1230; 1169; 1117; 1037; 978; 956; 901; 852
CFPOL	2.03	3336; 2921; 1612; 1412; 1230; 1095; 1035; 644

## Data Availability

The original contributions presented in this study are included in the article and/or the Supplementary Material. Further inquiries may be directed to the corresponding authors.

## Supplementary Materials

The following supporting information can be downloaded at: https://www.mdpi.com/article/10.3390/antiox14121403/s1, Figure S1: FT-IR spectra of CCFPOL. Figures S2–S12: NMR spectra of fractions and extracts from *C. cervicornis*. Figure S13: Cytotoxicity of extracts and fractions in 3T3 cells. Table S1: GC–MS data of compounds identified in extracts and fractions of *C. cervicornis*. Tables S2–S4: ^1^ H NMR data of selected diterpenes identified in extracts and fractions from *C. cervicornis.* Table S5: TPC and protein concentration in CCFPOL. Refs. [93,94,95,96,97,98,99,100,101,102] are cited in the Supplementary Materials.

## Abbreviations

The following abbreviations are used in this manuscript:

6-OHDA	6-Hydroxidopamine
ANOVA	Analysis of variance
AD	Alzheimer’s disease
ASD	Autism spectrum disorder
BBB	Blood–brain barrier
CO_2_	Carbon dioxide
DCM	Dichloromethane
DMEM	Dulbecco’s modified Eagle medium
ECCH	Hydroethanolic extract
ECCD	Dichloromethane extract
FBS	Fetal bovine serum
FT-IR	Fourier-Transform Infrared
GC-MS	Gas Chromatography-Mass Spectrometry
H_2_O_2_	Hydrogen peroxide
JC-1	5,5′,6,6′-tetrachloro-1,1′,3,3′-tetraethylbenzimi- dazolylcarbocyanine iodide
MMP	Mitochondrial membrane potential
MTT	3-(4,5-dimethylthiazol-2yl)-2,5-diphenltetrazolium bromide
NMR	Nuclear Magnetic Resonance
PD	Parkinson Disease
ROS	Reactive oxygen species
SH-SY5Y	Human neuroblastoma cell line
