# Supplementary material for: Chemical Characterization and Bio-Screening of Neuroprotective Potential of Brazilian Brown Seaweed Canistrocarpus cervicornis in 6-OHDA-Induced Neurotoxicity Model
Source: Antioxidants (Basel). 2025 Nov 25;14(12):1403. doi: 10.3390/antiox14121403 (PMC12729634; doi:10.3390/antiox14121403)
Supplement: Supplementary file 1 [file antioxidants-14-01403-s001.zip › antioxidants-3919872-supplementary.pdf]

Supplementary Data

**Figure S1**	FT-IR spectra of polysaccharide fraction (CCFPOL) in Kbrvmax/cm^{-1}.
**Figure S2-8**	1H NMR spectra of fractions from *Canistrocarpus cervicornis* (500.0 MHz, $CDCl_3$).
**Figure S9-12**	1H NMR spectra and COSY spectra of extracts from *Canistrocarpus cervicornis* (500.0 MHz, $CDCl_3$).
**Figure S13**	Cytotoxicity of *Canistrocarpus cervicornis* extracts and fractions (1-100 μg/mL; 24 h) on 3T3 cells.
**Table S1**	Retention time (RT), fragmentation data (*m/z*) and relative abundance (%) for chemical compounds identified in extracts and fractions of *Canistrocarpus cervicornis* by gas chromatography-mass spectrometry.
**Table S2-3**	^{1}H NMR data of major diterpenes identified in ECCH and ECCD ($CDCl_3$, 500 MHz, *J* in Hz).
**Table S4**	^{1}H NMR characteristic signals of selected diterpenes identified in *Canistrocarpus cervicornis* fractions (CCF1–2, CCF3, CCF4, CCF5–6, CCF7, CCF8–10, and CCF11–15).
**Table S5**	Quantification of total phenolic content (TPC) and protein concentration in the water-soluble fraction (CCFPOL) at 100 μg/mL of the brown seaweed *Canistrocarpus cervicornis*.

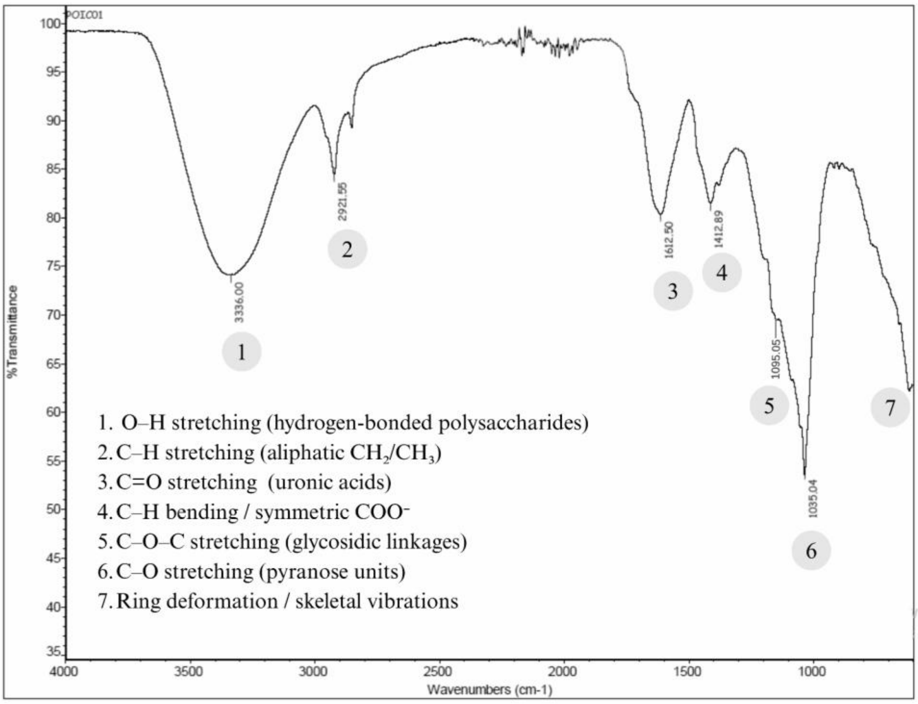

Figure S1. FT-IR spectra of the polysaccharide fraction (CCFPOL) in KBrv_{max}/cm^{-1}.

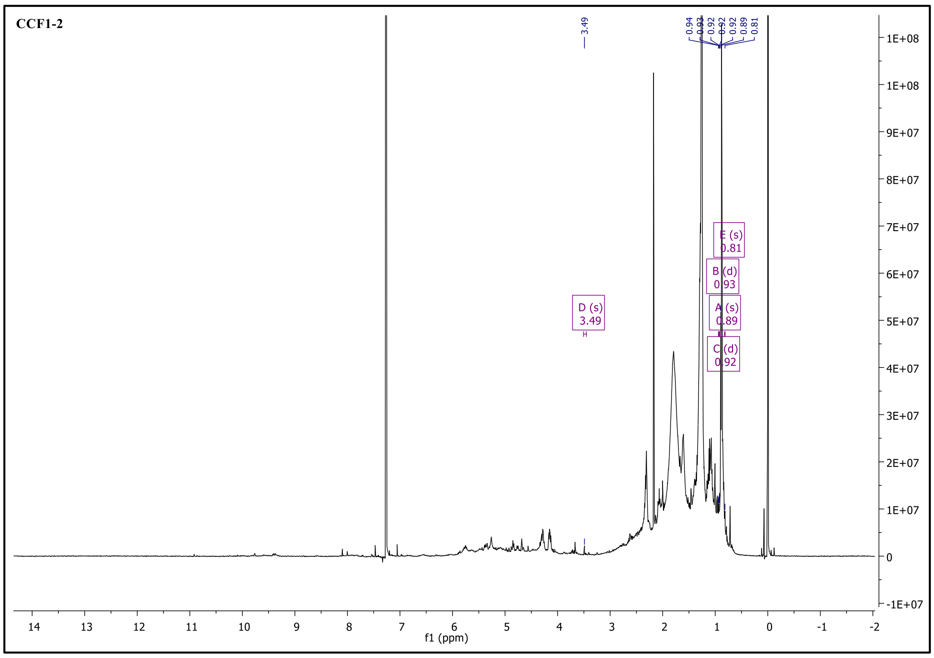

Figure S2. ^{1}H NMR spectra of CCF1-2 from *Canistrocarpus cervicornis* (500.0 MHz, $CDCl_3$).

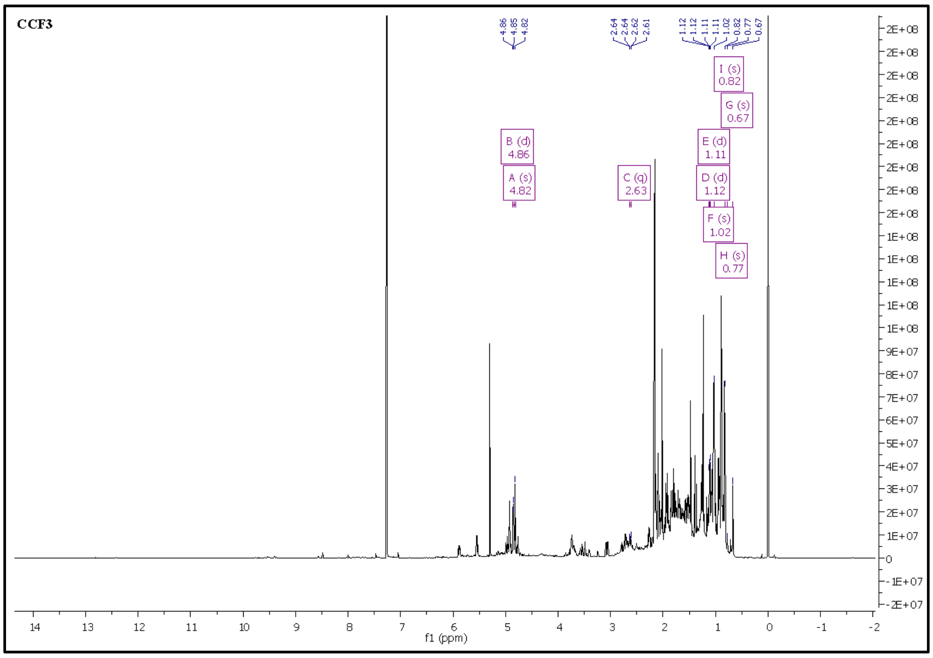

Figure S3. ^{1}H NMR spectra of CCF3 from *Canistrocarpus cervicornis* (500.0 MHz, $CDCl_3$).

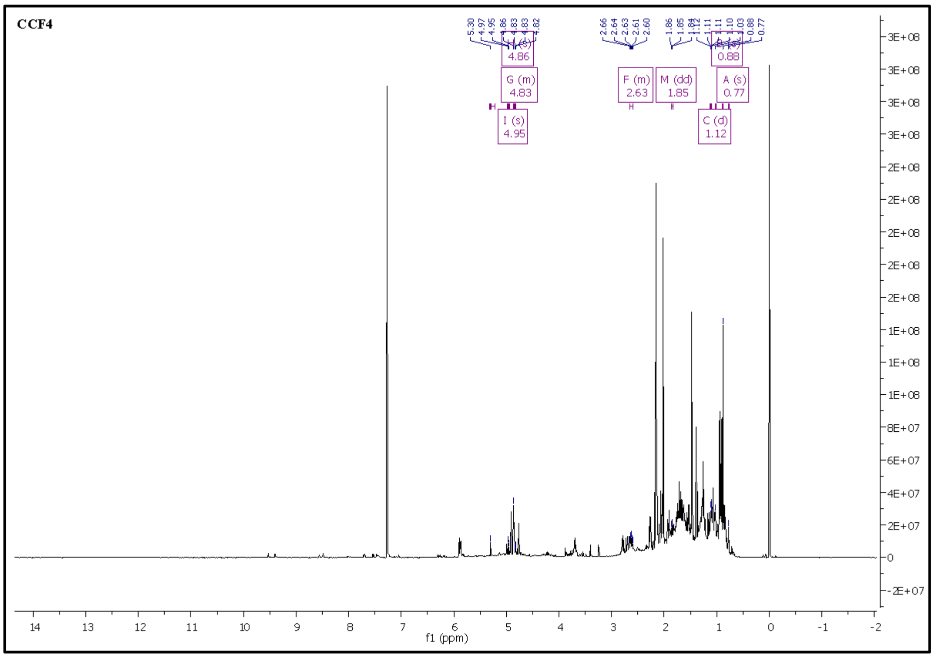

Figure S4. ^{1}H NMR spectra of CCF4 from *Canistrocarpus cervicornis* (500.0 MHz, $CDCl_3$).

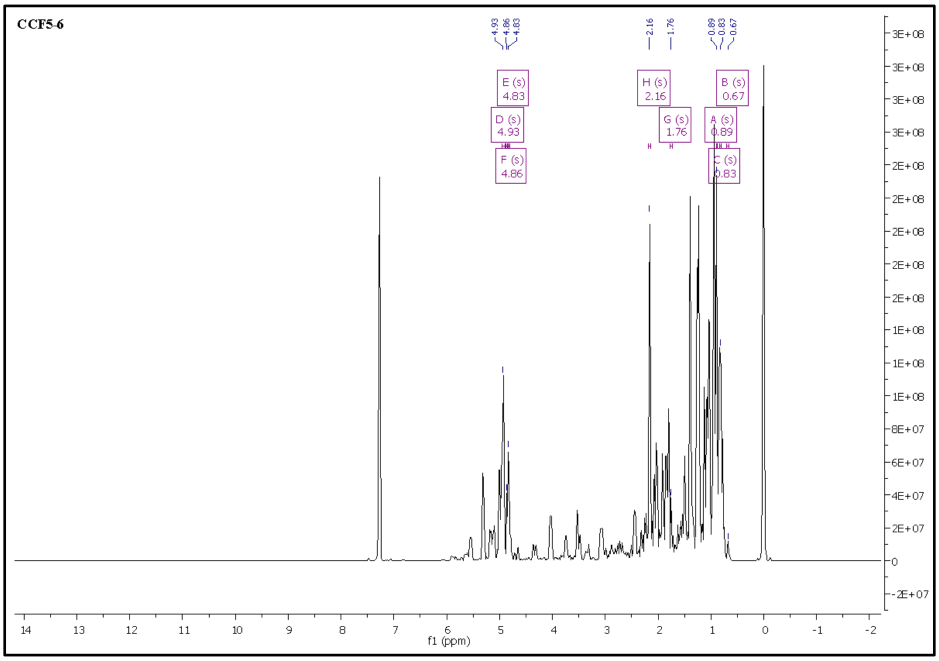

Figure S5. ^{1}H NMR spectra of CCF5-6 from *Canistrocarpus cervicornis* (500.0 MHz, $CDCl_3$).

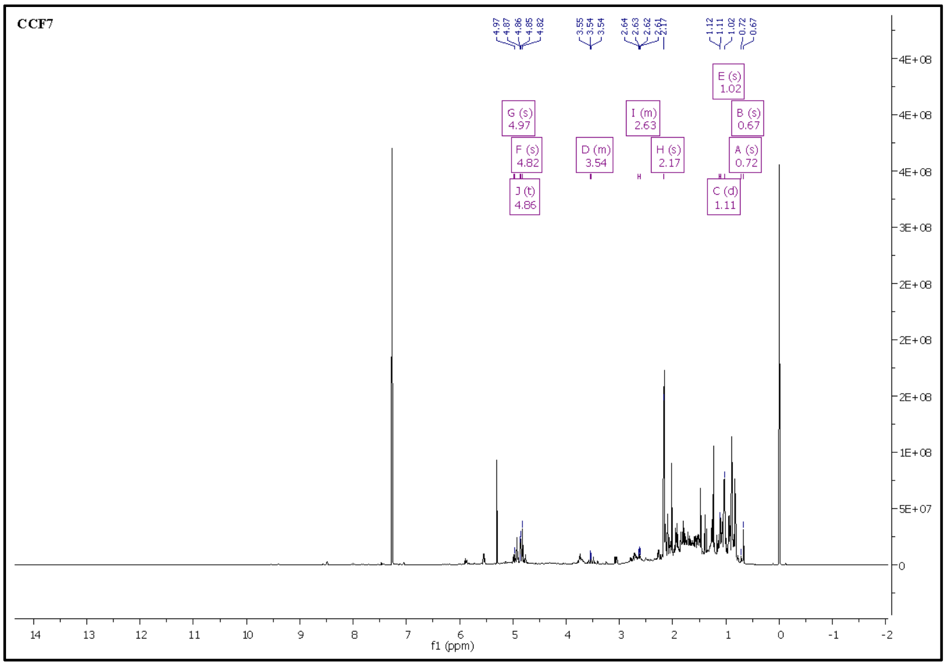

Figure S6. ^{1}H NMR spectra of CCF7 from *Canistrocarpus cervicornis* (500.0 MHz, $CDCl_3$).

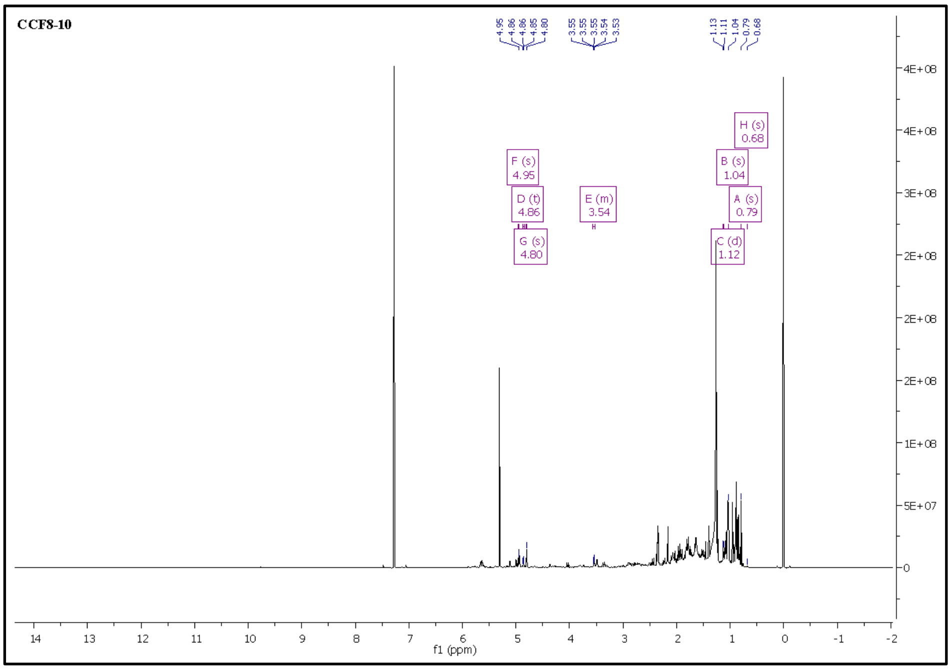

Figure S7. ^{1}H NMR spectra of CCF8-10 from *Canistrocarpus cervicornis* (500.0 MHz, $CDCl_3$).

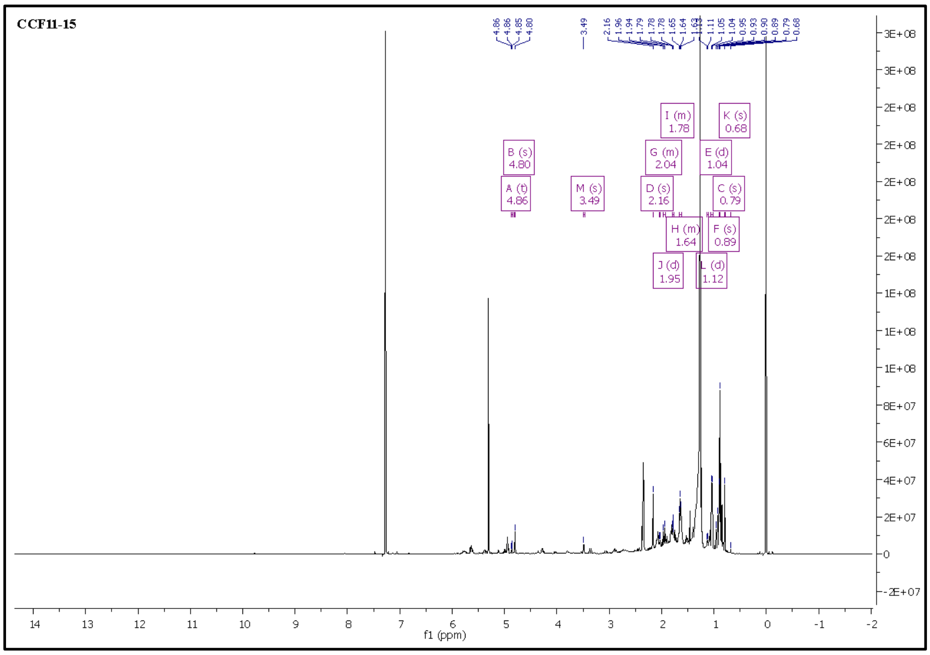

Figure S8. ^{1}H NMR spectra of CCF11-15 from *Canistrocarpus cervicornis* (500.0 MHz, $CDCl_3$).

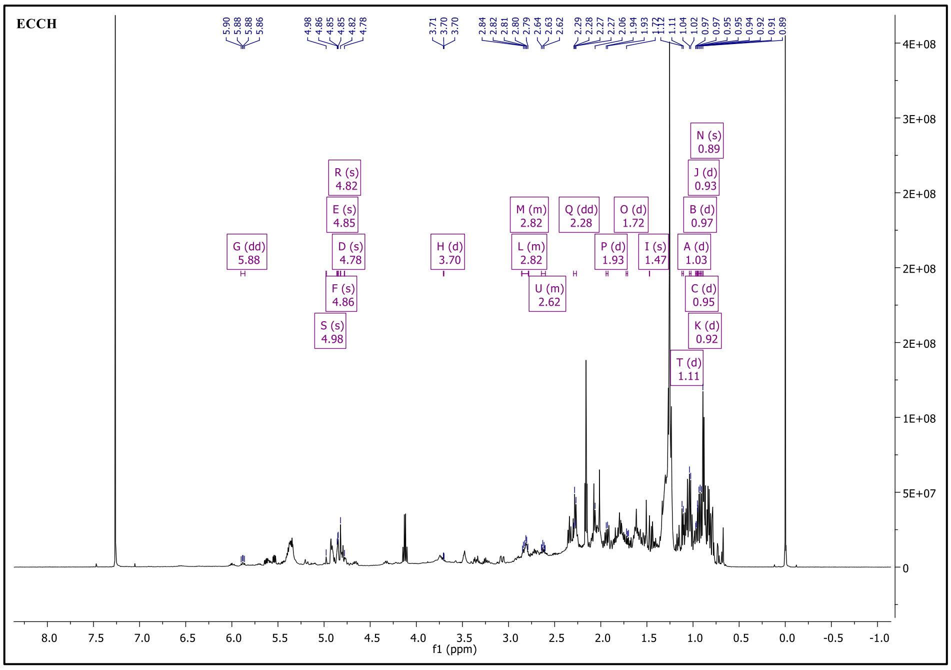

Figure S9. ^{1}H NMR spectra of hydroethanolic extract (ECCH) from *C. cervicornis* in $CDCl_3$ (500 MHz).

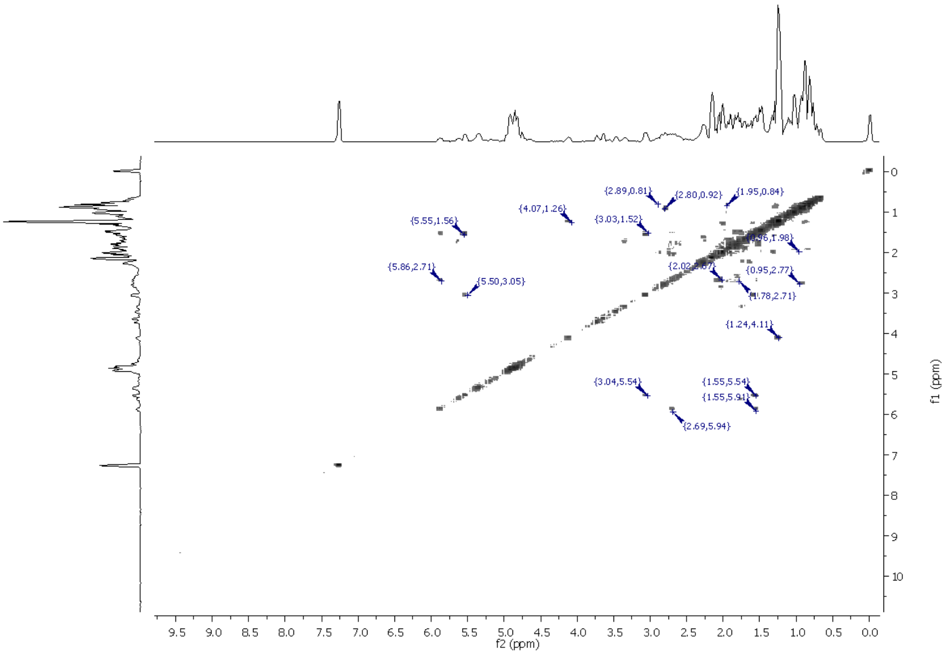

Figure S10. COSY spectrum of hydroethanolic extract (ECCH) from *C. cervicornis* in $CDCl_3$ (500 MHz).

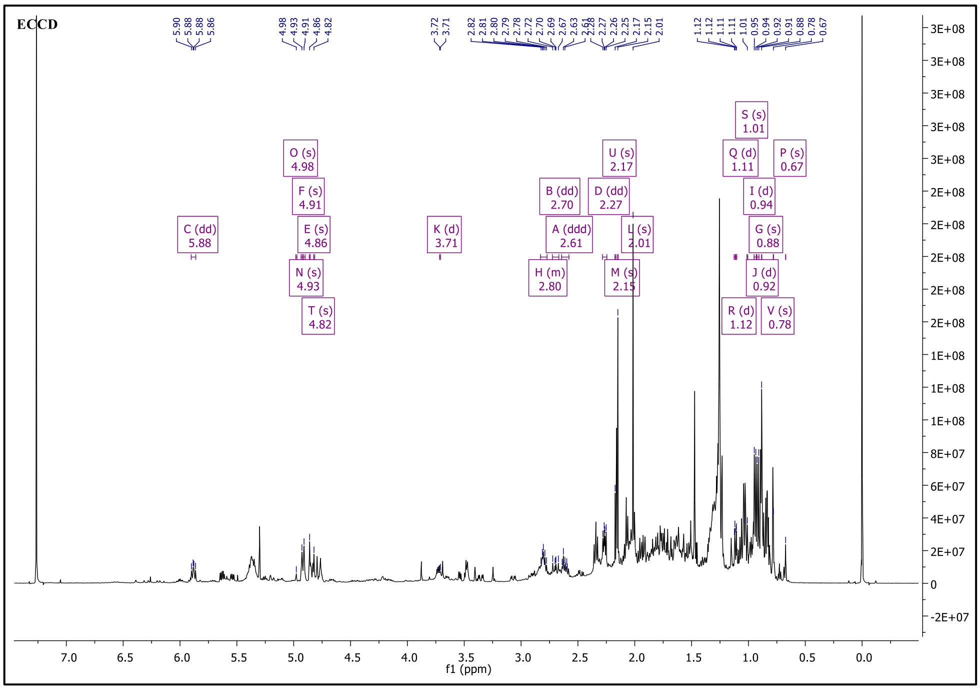

Figure S11. ^{1}H NMR spectra of dichloromethane extract (ECCD) from *C. cervicornis* in $CDCl_3$ (500 MHz).

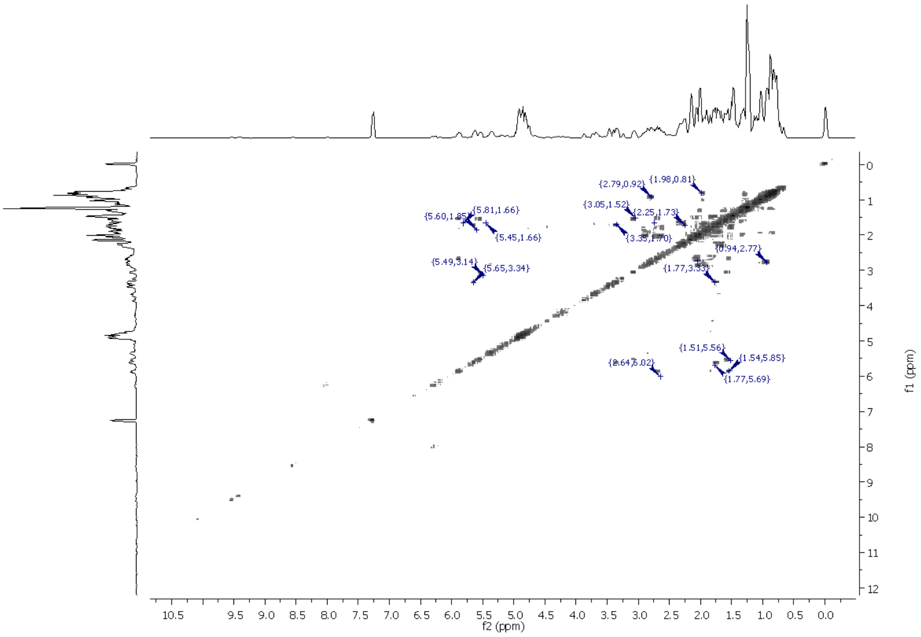

Figure S12. COSY spectrum of dichloromethane extract (ECCD) from *C. cervicornis* in $CDCl_3$ (500 MHz).

Table S1. Retention time (RT), fragmentation data (*m/z*) and relative abundance (%) for chemical compounds identified in extracts and fractions of *Canistrocarpus cervicornis* by gas chromatography-mass spectrometry.

**Compound**	**Class**	**RT**	**[M⁺]**	**Molecular formula**	**Fragments**	**Relative Abundance (%)**									
						**ECCD**	**ECCH**	**F1-2**	**F3**	**F4**	**F5-6**	**F7**	**F8-10**	**F11-15**	**Ref**
Tridecanoic acid	FA	13.235	214	$C_{13}H_{26}O_2$	214, 185, 171, 129, 115, 71, 73 (base peak), 60, 55, 57, 43, 41	0.21	-	20.80	-	-	-	-	-	-	[93]
α-linolenic acid	FA	23.034	278	$C1_{8}H_{30}O_2$	197, 107, 105 (base peak), 108, 91, 95, 93, 71, 67 55, 43	0.44	-	0.47	-	-	-	-	-	-	[94]
Stearic acid	FA	25.473	284	$C_{18}H_{36}O_2$	284, 270, 267, 255, 87, 91, 74 (base peak), 43	0.47	-	0.44	-	-	-	-	-	-	[95]
4,14-dihydroxy-dolastane-1(15),8-diene (Amijiol)	D	28.525	304	$C_{20}H_{32}O_2$	304, 286, 343, 149, 91, 55, 43 (base peak)	2.93	-	-	4.46	6.97	-	1.98	-	-	[96,97]
4-acetoxy-9,14-dihydroxy-dolastane-1(15),7-diene	D	28.644	362	$C_{22}H_{34}O_4$	362, 345, 344, 326, 319, 301, 266, 258, 251, 242, 241, 223, 217, 159, 157, 119, 93, 79, 43 (base peak), 41	2.913	-	-	-	-	-	-	-	-	[98,63]
4,9-dihydroxy-dolastane-1(15),7-diene	D	31.116	320	$C_{20}H_{32}O_2$	320, 302, 292, 277, 259, 229, 221, 217, 203, 145 (base peak), 119, 91, 55, 43	1.997	2.286	0.848	-	-	-	-	-	-	[32,63]
4,7,14-trihydroxy-dolastane-1(15),7-diene	D	31.798	320	$C_{20}H_{32}O_3$	320, 302, 284, 277, 266, 259, 241(base peak), 223, 199, 157, 149, 119, 105, 91, 55, 43	0.34	-	17.03	-	-	-	-	-	-	[97]
4,14-dihydroxy-dolastane-1(15),7,9-triene	D	31.974	302	$C_{20}H_{30}O_2$	302, 284, 263, 251, 241, 223, 209, 207, 157, 149, 133, 121,	2.93	-	-	4.46	6.97	-	1.98	-	-	[51]

					119, 105, 91, 77, 69, 55, 43										
4-acetoxy-14-hydroxy-dolastane-1(15),7,9-triene	D	32.901	344	$C_{22}H_{32}O_3$	344, 326, 301, 284, 266, 251, 243, 223, 149, 91, 55, 43 (base peak)	1.63	0.30	-	-	-	-	-	-	-	[98]
14-hydroxy-5-methyl-hydroxy-dolastane-1(15),8-diene	D	33.544	304	$C_{20}H_{32}O_2$	304, 284 (base peak), 241, 133, 119, 91, 55, 43	0.94	2.35	-	-	-	40.92	-	-	-	[63,99]
14-acetoxy-5-methyl-acetoxy-dolastane-1(15),8-diene	D	33.854	346	$C_{22}H_{34}O_3$	346, 326, 251, 133, 105, 91, 43 (base peak)	0.99	2.35	-	26.87	-	-	14.71	-	-	[63,99]
4-acetoxy-7,14-dihydroxy-dolastane-1(15), 8-diene	D	34.247	362	$C_{22}H_{34}O_4$	362, 344, 302, 284, 259, 241, 229, 149, 133, 121, 105, 91, 81, 69, 55, 43	2.913	-	-	39.25	34.16	10.49	14.90	-	-	[36,99]
7-acetoxy-4,14-dihydroxy-dolastane-1(15), 8-diene	D	35.392	362	$C_{22}H_{34}O_4$	362, 344, 302, 284, 259, 241, 229, 149, 133, 121, 105, 91, 81, 69, 55, 43	2.933	8.263	-	-	44.90	-	-	-	-	[36,100]
10ß-acetoxy-8,9-epoxy-14-hydroxy-7-oxodolastene	D	38.478	376	$C_{22}H_{32}O_5$	378, 360, 302, 284, 257, 149, 107, 91, 55, 43 (base peak)	4.987	6.27	-	-	-	-	-	-	-	[38]
4,7-diacetoxy-14-hydroxy-dolastane-1,9-diene	D	39.168	404	$C_{24}H_{36}O_5$	404, 386, 344, 326, 301, 288, 284, 266, 241, 223, 177, 149, 133, 121, 105, 93, 91, 73, 55, 43	10.43	12.52	-	-	-	-	-	-	1.73	[100]
Amijidictyol	D	40.567	420	$C_{24}H_{36}O_6$	420, 358, 298, 255, 149, 135, 91, 43 (base peak)	-	26.96	-	-	-	-	-	-	-	[101]
Isolinearol	S	36.206	336	$C_{20}H_{30}O_3$	336, 318, 316, 275, 219	2.45	0.80	-	-	-	-	47.46	64.79	77.75	[32,51]
Linearol acetate	S	37.257	378	$C_{22}H_{34}O_5$	378, 318, 303, 280, 278, 257, 247, 245, 238, 233, 227, 219 (base peak), 194, 173, 159, 157, 149, 147, 133, 125, 119, 71, 55	2.45	3.25	2.18	-	-	-	4.80	1.17	5.06	[51]

Isolinearol acetate	S	37.511	378	$C_{22}H_{34}O_5$	378, 319, 302, 280, 278, 257, 247, 245, 238, 233, 227, 219, 194, 173, 159, 157, 149, 147, 133, 125, 119, 71, 55	18.39	12.53	0.94	3.52	-	7.26	7.82	3.96	14.21	[32,51]
Dichotenol B	S	39.516	394	$C_{22}H_{34}O_6$	394, 376, 189, 91, 71, 43 (base peak)	14.08	6.33	-	2.89	3.49	1.73	-	21.12	1.25	[102]
Not identified compounds (NI)						25.59	15.80	56.55	18.56	3.51	27.60	6.25	8.96	0.00	-

Table S2. ^{1}H NMR data of major diterpenes identified in ECCH ($CDCl_3$, 500 MHz, *J* in Hz).

Position	4,7-diacetoxy-14-hydroxy-dolastane-1,9-diene[a] δ H (nº H; *m*; J Hz)	Amijidictyol[b] δ H (nº H; *m*; J Hz)	Isolinearol acetate[c] δ H (nº H; *m*; J Hz)
7	5.88 (1H, *dd*, *J* = 10.8, 7.3)	nd	nd
10	2.28 (1H, *dd*, J = 9.0, 6.0 Hz)	5.88 (1H, *dd*, J= *10.8*, *7.3*)	nd
13	1.93 (1H, *d*, J= 7.0 Hz)	nd	nd
15	4.85 (1H, *s*)/ 4.93 (1H, *s*)	4.86 (1H, *sl*)/4.78 (1H, *s*)	4.82 (1H, *sl*)/ 4.98 (1H, *sl*)
16	0.89 (1H, *s*)	0.70 (3H, *s*)	0.79 (3H, *s*)
17	2.79 (1H, *m*)	2.78 (1H, *s*)	2.63 (1H, *m*)
18	0.92 (1H, *d*, J= 6.3 Hz)	0,95 (3H, d, 6.9)	1.11 (*d*, J= 6.9 Hz, 3H)
19	0.94 (1H, *d*, J= 6.3 Hz)	1.03 (1H, *d*, J= 6.7 Hz)	1.11 (*d*, J= 6.9 Hz, 3H)
20	1.47 (3H, *s*)	1.53 (3H, *s*)	1.01 (1H, s)
4-OH	-	3,89 (1H, *d*, 7.6)	-
4-OAc	2.15 (3H, *s*)	-	2.17 (1H, s)
7- OAc	2.08 (3H, *s*)	2.06 (3H, sl)	-
10- OAc	-	2.04 (3H, *s*)	nd
14-OH	3.70 (1H, *d*, J = 2.3 Hz)	n.d	-

Chemical shift/ppm (multiplicity; coupling constant); nd: not detected. References: [a]Kelecom; Teixeira [36]; [b] Ochi et al. [101]; [c]Teixeira et al. [51].

Table S3. ^{1}H NMR data of major diterpenes identified in ECCD ($CDCl_3$, 500 MHz, *J* in Hz).

Position (C/H)	4,7-diacetoxy-14-hydroxy-dolastane-1,9-diene[a]	Isolinearol[b]	Isolinearol acetate[b]
1	-	2.80 *d* (1.0)	-
2	2.62 (1H, *m*)	nd	-
6	2.70 (1H, *dd*, *J* = 14.1, 11 Hz)	nd	nd
7	5.88 (1H, d, J= 11, 7.0 Hz)	nd	nd
10	2.26 (1H, *dd*, 9.0, 6.0 Hz)	nd	nd
13	nd	nd	nd
14	-	-	-
15	4.86 (1H, *s*) e 4.91 (1H, *s*)	4.83 (1H, *s*) e 4.98 (1H, *s*)	4.82 (1H, s)/ 4,98 (1H, s)
16	0.88 (3H, *s*)	0.67 (1H, *s*)	0.78 (3H, d)
17	2.80 (1H, *m*)	2.63 (1H, *m*)	2.62 (1H, *m*)
18	0.91 (3H, *d*, J= 6.8 Hz)	1.11 (3H, *d*, 6.9 Hz)	1.11 (3H, *d*, 6.9 Hz)
19	0.94 (3H, *d*, J= 6.8 Hz)	1.12 (3H, *d*, 6.9 Hz)	1.12 (3H, *d*, 6.9 Hz)
20	1.47 (3H, *s*)	1.01 (3H, s)	1.01 (1H, s)
4-OH	-	3.54 (1H, *m*)	2.17 (1H, s)
8-OH	-	3.54 (1H, *m*)	nd
4-OAc	2.15 (3H, *s*)	-	-
7-OAc	2.01 (3H, *s*)	-	-
14-OH	3.71 (1H, *d*, J= 2.0)	-	-

Chemical shift/ppm (multiplicity; coupling constant); nd: not detected. References: [a]Kelecom; Teixeira [36]; [b] Ochi et al. [101].

Table S4. ^{1}H NMR characteristic signals of selected diterpenes identified in *Canistrocarpus cervicornis* fractions (CCF1–2, CCF3, CCF4, CCF5–6, CCF7, CCF8–10, and CCF11–15) based on literature data [36,51,61] ($CDCl_3$, 500 MHz, *J* in Hz). In the table, x indicates signal detected; nd indicates not detected.

Position (C/H)	**4,14-dihydroxy-dolastane-1(15),8-diene (Amijiol)** De Oliveira et al. [61]	CCF1-2	CCF3	CCF4	CCF5-6	CCF7	CCF8-10	CCF11-15
4	3.40 (1H, *t*, J= 4.0)	nd	nd	x	x	x	nd	nd
15	4.84 (1H, *bs*)	nd	x	x	x	nd	x	x
16	0.75 (3H, *s*)	nd	x	x	nd	nd	nd	nd
18	0.92 (3H, *d*, J= 6.9)	nd	x	x	x	x	+	nd
19	0.97 (3H, *d*, J= 6.9)	nd	nd	x	x	x	nd	nd
20	1.37 (3H, *s*)	nd	x	x	x	x	x	x
	**4,14-dihydroxy-dolastane-1(15),7,9-triene** De Oliveira et al. [61]	**CCF1-2**	**CCF3**	**CCF4**	**CCF5-6**	**CCF7**	**CCF8-10**	**CCF11-15**
4	3.48 (1H, *s*)	x	x	nd	x	x	x	nd
7	5.44(1H, *dd*, J= 9.5, 4.2)	nd	x	nd	x	x	nd	nd
10	4.78 (1H, *bs*)	nd	x	x	x	nd	x	x
15	4.90 (1H, *bs*)	nd	nd	x	nd	x	x	nd
16	0.82 (3H, s)	x	x	x	x	x	x	x
18	1.05 (3H, d, J= 6.9)	nd	x	x	x	x	nd	nd
19	1.12 (3H, d, J= 6.9)	nd	x	x	nd	x	nd	nd
20	1.35 (3H, *s*)	nd	x	x	nd	x	x	nd
	**4,7-diacetoxy-14-hydroxy-dolastane-1,9-diene** Kelecom; Teixeira [36]	**CCF1-2**	**CCF3**	**CCF4**	**CCF5-6**	**CCF7**	**CCF8-10**	**CCF11-15**
7	5.88 (1H, *dd*, *J* = 10.8, 7.3)	nd	x	x	x	x	x	nd
15	4.86 (1H, *s*) e 4.91 (1H, *s*)	nd	x	x	x	x	x	x
16	0.89 (1H, *s*)	x	nd	x	x	x	x	x
17	2.70 (1H, *dd*, J = 14.1, 11 Hz)	nd	nd	nd	x	x	nd	nd
18	0.92 (1H, *d*, J= 6.3 Hz)	x	x	nd	x	x	nd	x
19	0.94 (1H, *d*, J= 6.3 Hz)	x	x	nd	x	x	nd	x
20	1.47 (3H, *s*)	nd	x	x	nd	nd	x	x
4-OAc	2.15 (3H, *s*)	nd	x	x	nd	nd	x	x
7- OAc	2.08 (3H, *s*)	nd	x	x	nd	nd	x	x
	**Isolinearol acetate** Teixeira et al. [51]	**CCF1-2**	**CCF3**	**CCF4**	**CCF5-6**	**CCF7**	**CCF8-10**	**CCF11-15**
15	4.82 (1H, s)/ 4,96 (1H, s)	nd	x	nd	x	x	x	x
16	0.78 (3H, d)	nd	x	x	x	x	x	x
17	2.62 (1H, *m*)	nd	x	x	nd	x	x	x
18	1.11 (3H, *d*, 6.9 Hz)	nd	x	x	nd	x	x	x
19	1.11 (3H, *d*, 6.9 Hz)	nd	x	x	nd	x	x	x
20	1.02 (1H, s)	nd	x	x	nd	x	x	x
4-OH	2.18 (1H, s)	nd	x	x	nd	x	x	x
	**Isolinearol** Teixeira et al. [51]	**CCF1-2**	**CCF3**	**CCF4**	**CCF5-6**	**CCF7**	**CCF8-10**	**CCF11-15**
15	4.83 (1H, *s*) e 4.98 (1H, *s*)	nd	x	nd	x	x	x	x
16	0.67 (1H, *s*)	nd	x	x	x	x	x	x
17	2.63 (1H, *m*)	nd	x	x	x	x	x	x
18	1.11 (3H, *d*, 6.9 Hz)	nd	x	x	x	x	x	x
19	1.12 (3H, *d*, 6.9 Hz)	nd	x	x	x	x	x	x
20	1.01 (3H, s)	nd	x	x	x	x	x	x
4-OH	3.54 (1H, *m*)	nd	x	x	x	x	x	x
8-OH	3.54 (1H, *m*)	nd	x	x	x	x	x	x

δ H (nº H; m; J Hz); Chemical shift/ppm (multiplicity; coupling constant).

Table S5. Quantification of total phenolic content (TPC) and protein concentration in the water-soluble fraction (CCFPOL) at 100 µg/mL of the brown seaweed *Canistrocarpus cervicornis*.

**Assay**	**TPC**[a]	Protein[b]
Concentration	ND	ND
Y=aX+b	(0.6528 ± 0.0046)X + (0.0530 ± 0.0016)	(0.03104 ± 0.00092)X + (0.4762 ± 0.0139)
R^2	0.9995 ± 0.0002	0.9981 ± 0.0031

[a]TPC: mg of gallic acid equivalents/g of CCFPOL (mg GAE/g), based on a gallic acid calibration curve. [b]Protein content: mg of Protein/g of CCFPOL (mg Protein/g) based on a albumin calibration curve. The linear regression equations and coefficients of determination (R^2) are presented as mean ± standard error of the mean (SEM), based on three independent experiments (n=3). ND: not detected.

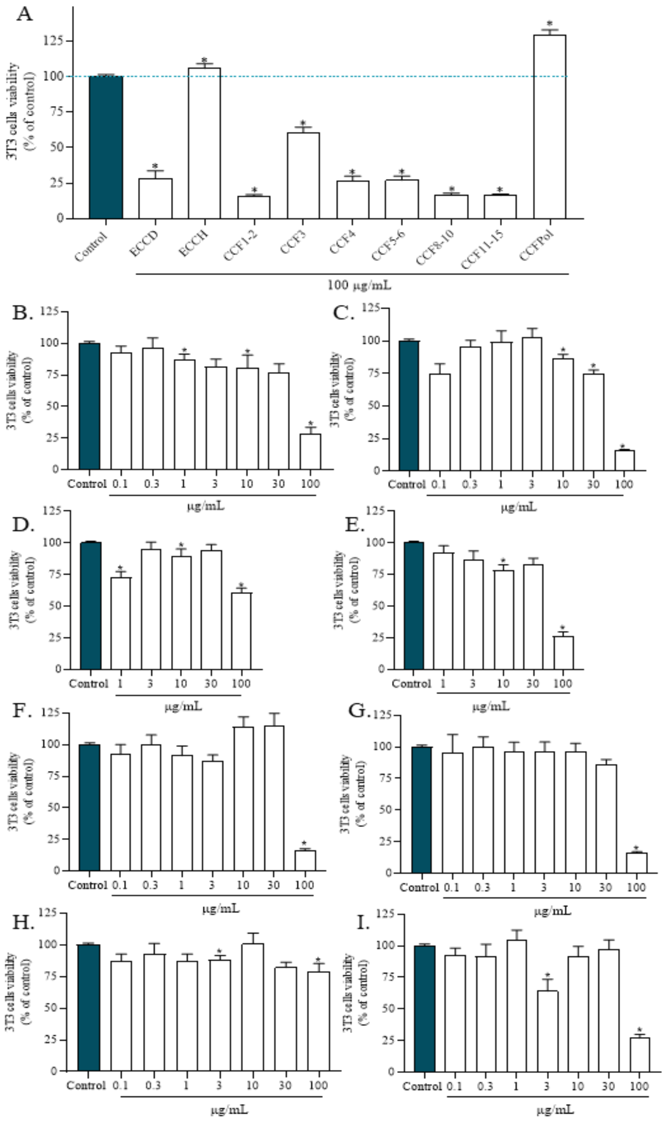

Figure S11. Cytotoxicity of *Canistrocarpus cervicornis* extracts and fractions (1-100 µg/mL; 24 h) on 3T3 cells. **A**. Cytotoxicity at 100 µg/mL of all samples; **B**. Dichloromethane extract (ECCD); **C**. CCF1-2; **D.** CCF3; **E**. CCF4; **F**. CCF5-6; **G**. CCF7; **H**. CCF8-10; **I**. CCF11-15. The values in each column represent the mean ± standard error of the mean (SEM) of 3 or 4 independent experiments. The symbol (*) represents significant differences (p < 0.05; Paired Student's t-test). Vehicle (DMSO or H_2O) was considered to be 100% viable.

References

32. Obando, J.M.C.; Dos Santos, T.C.; Fortes, R.D.R.; Bernardes, M.; Nascimento, N.; Villaça, R.C.; Teixeira, V.L.; Barbarino, E.; Cavalcanti, D.N. Influence of laboratory conditions on two species Dictyotaceae family (Phaeophyceae): Diterpenoid profile and physiological response. *Front Mar Sci* **2023**, 10. https://doi.org/10.3389/fmars.2023.1150719.
36. Kelecom, A.; Teixeira, V.L. Dolastane Diterpenes from the Marine Brown Alga *Dictyota cervicornis*. *Phytochemistry* **1988**, *27*, 2907–2909. https://doi.org/10.1016/0031-9422(88)80686-1
38. Bianco, E.M.; Francisco, T.M.; Pinheiro, C.B.; Azeredo, R.B.; Teixeira, V.L.; Pereira, R.C. 10b-Acetoxy-8a,9a-epoxy-14b-hydroxy-7-oxodolastane-ANew Diterpene Isolated from the Brazilian Brown Macroalga *Canistrocarpus cervicornis*. *Helv Chim Acta* **2015**, 98, 785-794. https://doi.org/10.1002/hlca.201400321.
51. Teixeira, V.L.; Tomassini, T.; Fleury, B.G.; Kelecom, A. Dolastane and Secodolastane Diterpenes from the Marine Brown Alga, *Dictyota cericornis*. *J Nat Prod* **1986**, *49*, 570–575. https://doi.org/10.3389/fmars.2023.1150719.
61. De Oliveira, A.S.; Cavalcanti, D.N.; Bianco, E.M.; De Paula, J.C.; Pereira, R.C.; Valentin-Yoneshigue, Y.; Teixeira, V.L. Chemical Composition of Diterpenes from the Brown Alga *Canistrocarpus cervicornis* (Dictyotaceae, Phaeophyceae). *Nat Prod Commun* **2008**, *3*. https://doi.org/10.1177/1934578X0800300913.
63. Obando, J.M.C; Dos Santos, T.C.; Bernardes, M.; Nascimento, N.; Villaça, R.C.; Barbarino, E.; Teixeira, V.L.; Cavalcanti, D.N. Diterpene Biosynthesis of Seaweed *Canistrocarpus cervicornis*: A Potential Method by Diterpene Obtention in Brown Algae Using Aqueous Extract as an Enzyme Source. *J. Of Aquac & Fish* **2024**, *87*, 1-7. https://doi.org/10.24966/AAF-5523/100087.
93. NIST Mass Spectrometry Data Center NIST MS (352605) Tridecanoic Acid. Available online: https://webbook.nist.gov/cgi/cbook.cgi?ID=C638539&Mask=200 (accessed on 4 September 2025).
94. NIST Mass Spectrometry Data Center NIST MS (333201) 9,12,15-Octadecatrienoic Acid, (*Z,Z,Z*)-. Available online: https://webbook.nist.gov/cgi/cbook.cgi?Name=%CE%B1-Linolenic+acid&Units=SI&cMS=on&cGC=on (accessed on 4 June 2025).
95. NIST Mass Spectrometry Data Center NIST MS (290961) Octadecanoic Acid Available online: https://webbook.nist.gov/cgi/cbook.cgi?Name=Stearic+acid&Units=SI&cMS=on (accessed on 17 May 2025).
96. Ayyad, S.E.N.; Makki, M.S.; Al-Kayal, N.S.; Basaif, S.A.; El-Foty, K.O.; Asiri, A.M.; Alarif, W.M.; Badria, F.A. Cytotoxic and Protective DNA Damage of Three New Diterpenoids from the Brown Alga *Dictoyota dichotoma*. *Eur J Med Chem* **2011**, 46, 175–182. https://doi.org/10.1016/j.ejmech.2010.10.033.
97. Crews, P.; Klein, T.E.; Hogue, E.R.; Myers, B.L. Tricyclic Diterpenes from the Brown Marine Algae *Dictyota divaricata* and *Dictyota linearis*. *J Org Chem* **1982**, *47*, 811–815. https://doi.org/10.1021/jo00344a012.
98. Garcia, D.G.; Bianco, E.M.; Santos, M. da C.B. dos; Pereira, R.C.; Faria, M.V. de C.; Teixeira, V.L.; Burth, P. Inhibition of Mammal Na+K+ATPase by Diterpenes Extracted from the Brazilian Brown Alga *Dictyota cervicornis*. *Phytotherapy Research* **2009**, *23*, 943–947. https://doi.org/10.1002/ptr.2500.
99. Sun, H.H.; McConnell, O.J.; Fenical, W.; Hirotsu, K.; Clardy, J. Tricyclic Diterpenoids of the Dolastane Ring System from the Marine Alga *Dictyota divaricata*. *Tetrahedron* **1981**, *37*, 1237–1242. https://doi.org/10.1016/S0040-4020(01)92057-5
100. Bianco, E.M.; Francisco, T.M.; Pinheiro, B.C.; Azeredo, B.V.D.R.; Pereira, R.C. 4a-Acetoxyamijidictyol – A New Antifeeding Dolastane Diterpene from the Brazilian Brown Alga *Canistrocarpus cervicornis. Chem Biodivers* **2015**, *12*, 1665–1677. https://doi.org/10.1002/cbdv.201400410.
101. Ochi, M.; Watanabe, M.; Kido, M.; Ichikawa, Y.; Miura, I.; Tokoroyama, T. Amijidictyol, a New Diterpenoid from the Brown Seaweed Dictyota linearis: X-Ray Crystal and Molecular Structure. *Chem Lett* **1980**, *9*, 1233–1234. https://doi.org/10.1246/cl.1980.1233.
102. Ali, M.S.; Pervez, M.K.; Ahmed, F.; Saleem, M. Dichotenol-A, B and C: The C-16 Oxidized Seco-Dolastanes from the Marine Brown Alga *Dictyota dichotoma* (HUDS.) Lamour. *Nat Prod Res* **2004**, *18*, 543–549. https://doi.org/10.1080/14786410310001622059.
